# 8,8′-(Benzo[*c*][1,2,5]thiadiazole-4,7-diyl)bis(quinolin-4(1*H*)-one): a twisted photosensitizer with AIE properties†

**DOI:** 10.1039/d1ra06263h

**Published:** 2021-09-01

**Authors:** Emmanouil Broumidis, Callum M. S. Jones, Maria Koyioni, Andreas Kourtellaris, Gareth O. Lloyd, Jose Marques-Hueso, Panayiotis A. Koutentis, Filipe Vilela

**Affiliations:** Institute of Chemical Sciences, School of Engineering & Physical Sciences, Heriot-Watt University Edinburgh EH14 4AS UK F.Vilela@hw.ac.uk; Institute of Sensors, Signals and Systems, School of Engineering & Physical Sciences, Heriot-Watt University Edinburgh EH14 4AS UK; Department of Chemistry, University of Cyprus P.O. Box 20537 1678 Nicosia Cyprus; Joseph Banks Laboratories, School of Chemistry, University of Lincoln Brayford Pool Lincoln LN6 7TS UK

## Abstract

A new benzothiadiazole (BTZ) luminogen is prepared *via* the Suzuki–Miyaura Pd-catalysed C–C cross-coupling of 8-iodoquinolin-4(1*H*)-one and a BTZ bispinacol boronic ester. The rapid reaction (5 min) affords the air-, thermo-, and photostable product in 97% yield as a yellow precipitate that can be isolated by filtration. The luminogen exhibits aggregated-induced emission (AIE) properties, which are attributed to its photoactive BTZ core and nonplanar geometry. It also behaves as a molecular heterogeneous photosensitizer for the production of singlet oxygen under continuous flow conditions.

## Abstract 2

A new benzothiadiazole (BTZ) luminogen is prepared *via* the Suzuki–Miyaura Pd-catalysed C–C cross-coupling of 8-iodoquinolin-4(1*H*)-one and a BTZ bispinacol boronic ester.

## Graphical abstract

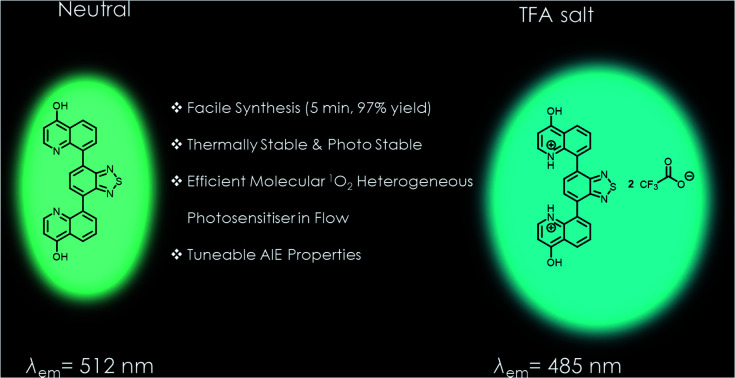

## Introduction

Photoluminescent materials such as organic dyes and fluorescent polymers have multiple applications in areas such as chemical sensing, organic electronics and bioimaging.^1^ Up until the dawn of the 21^st^ century, most organic luminescent materials were typically large, planar and highly conjugated systems. Unsurprisingly, these materials suffer from aggregation-caused quenching (ACQ) effects, owing to solid state face-to-face π stacking interactions, that fully or partially suppress the photoluminescent (PL) properties and thus, the intended functionality of these materials.^2^

In 2001, Tang and coworkers^3^ introduced a silole-based organic material which exhibited the opposite behaviour of known ACQ luminogens. Specifically, it was weakly emitting in solution but a strong emitter in the solid state. This unusual photophysical phenomenon is known as aggregation-induced emission (AIE), and there have been extensive efforts to uncover its underlying mechanism of action, as well as develop new photoactive materials with AIE properties.^4^ Most AIE active materials (AIEgens) have rigid, nonplanar geometries in contrast to the planar ACQ based materials. In solution, an AIEgen is subject to intermolecular rotation, vibration or stretching and, as such, any emissions occur mostly *via* non-radiative processes. In contrast, when in an aggregated or solid state, owing to their rigid nonplanar geometry, π–π stacking and intermolecular motions are limited. This leads to a significant enhancement of the corresponding photoluminescence quantum yield (PLQY), as radiative processes become more prevalent.^5^ While other mechanistic pathways exist, such as the twisted intramolecular charge transfer process (TICT)^6^ or crystallisation-induced emission enhancement (CIEE),^7^ the aforementioned restriction of intramolecular motion (RIM) process is dominant for most AIEgen materials.^4^ Even though in recent years there have been significant advancements in the development of new AIEgen materials, particularly in the organic light-emitting diode (OLED) industry,^8^ some of the challenges that remain include the need for tuneable and multifunctional AIEgens with properties such as broad excitation wavelength range, increased biocompatibility, and ability of generating reactive oxygen species (ROS).^5^ These characteristics are especially useful for synthesising AIEgens which can be used as probes for the elucidation of antibiotic mechanisms of action,^9^ or as potent agents for photodynamic therapy (PDT).^10^

One of the luminophores that has attracted the attention of researchers in recent years is the *S*,*N*-heterocycle 2,1,3-benzothiadiazole (BTZ). BTZ and its derivatives have an appealing set of properties which can make them useful building blocks for developing new photoactive materials.^11–13^ These include their biocompatibility, and their strong electron-withdrawing character that makes them useful partners in donor (D)–acceptor (A) or D–A–D photoactive systems as well as in organic semiconductor materials such as photovoltaics and OLEDs.^14^ As part of our work on new molecular and polymeric BTZ containing photoactive materials,^15–18^ we have developed a high yielding, scalable and rapid synthesis of a new quinolin-4-ol incorporating a central BTZ unit. Gratifyingly, this material has a helical, nonplanar geometry, and exhibits AIE and ROS generation properties.

## Results and discussion

### Synthesis and characterisation

**Scheme 1:**
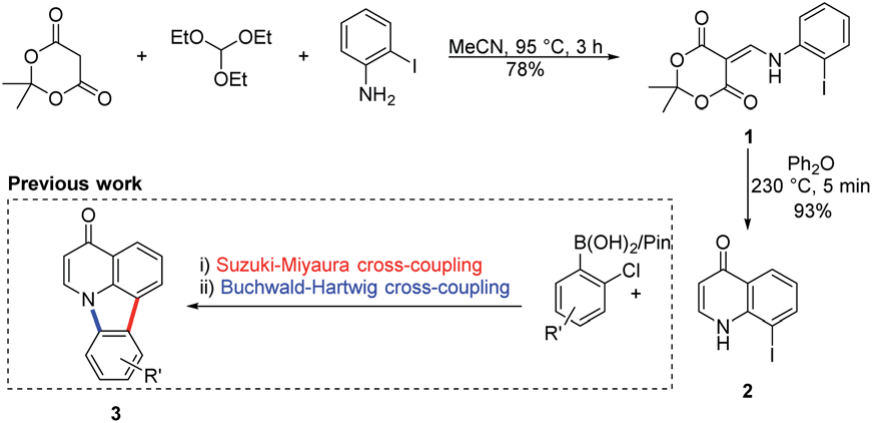
Synthetic pathway for and use of 8-iodoquinolin-4(1*H*)-one 2 to prepare deazacanthin-4-one 3.

The chemistry used to access the starting material, the antimicrobial core^19^ 8-iodoquinolin-4(1*H*)-one (2), was previously developed by Koutentis and coworkers to gain access to the natural product derivative deazacanthin-4-one 3 (Scheme 1).^20,21^

**Scheme 2:**
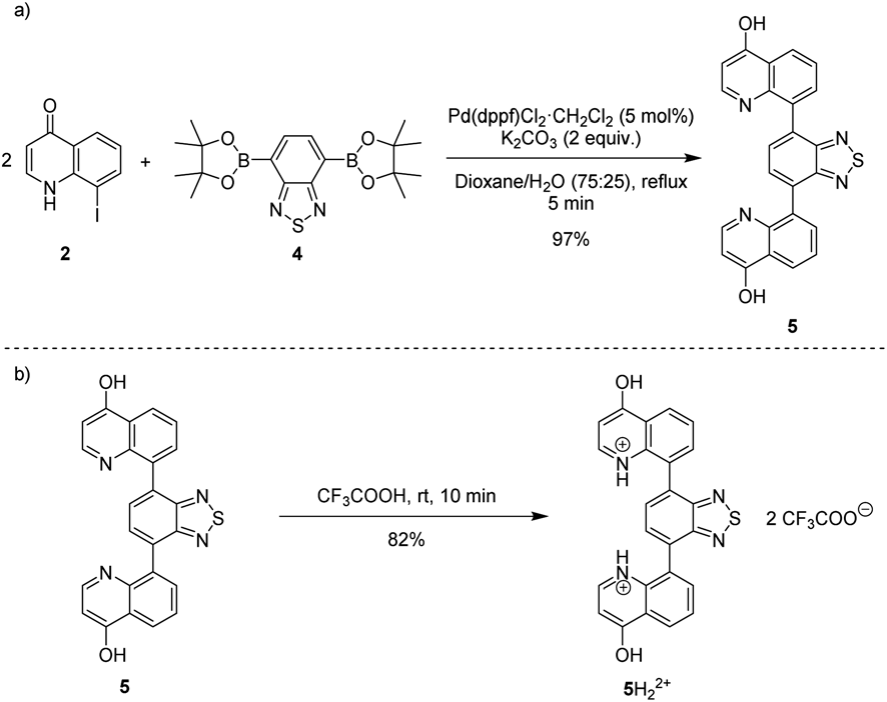
(a) Suzuki–Miyaura reaction of 8-iodoquinolin-4(1*H*)-one (2) with BTZ(Bpin)_2_4 to afford 8,8'-(benzo[*c*][1,2,5]thiadiazole-4,7-diyl)bis(quinolin-4-ol) (5). (b) Synthesis of 5H_2_^2+^ from 5.

**Fig. 1:**
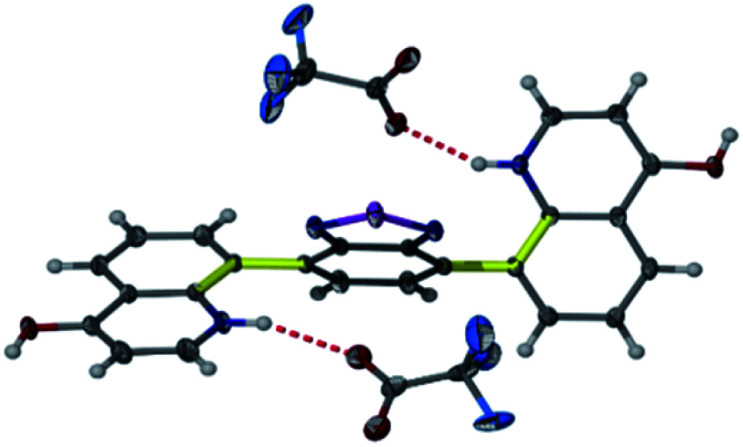
Structure of 5H_2_^2+^ shown as 50% probability ellipsoids. Hydrogen bonding of the TFA molecules to 5H_2_^2+^ highlights the protonation and enol tautomer of 5. Disorder of one of the triflate and both TFA molecules in the asymmetric unit are not shown for clarity. Yellow and thicker bonds indicate the torsion angle of 126.11(17)°, which represents the rotation of the two quinolinol groups to opposite sides of the BTZ.

Heating an equimolar mixture of triethylorthoformate, Meldrum's acid and 2-iodoaniline in MeCN under reflux conditions for 3 h, gives 5-{[(2-iodophenyl)amino]methylene}-2,2-dimethyl-1,3-dioxane-4,6-dione (1), thermolysis of which affords 8-iodoquinolin-4(1*H*)-one (2).^17^ In our prior work, quinolone 2 was reacted with 2-chloroarylboronic acids or pinacol esters in a two-step, C–C and C–N bond forming process to afford the desired deazacanthin-4-ones 3.^21^ Herein, quinolone 2 was reacted with 4,7-bis(4,4,5,5-tetramethyl-1,3,2-dioxaborolan-2-yl)benzo[*c*][1,2,5]thiadiazole [BTZ(Bpin)_2_] (4) to give the desired 8,8′-(benzo[*c*][1,2,5]thiadiazole-4,7-diyl)bis(quinolin-4-ol) (5) in up to 97% yield (Scheme 2). Product 5 precipitated from the reaction mixture and, following a simple filtration and subsequent washes with THF and EtOH, was collected as a yellow semi-crystalline powder without the need for further purification, as determined by LC-MS (ESI, S2.5 and S2.6†). Compound 5 exhibited very poor solubility in both polar and non-polar solvents (ESI, S2.7†); hot DMSO only sparingly dissolved the compound and was not suitable for obtaining satisfactory ^13^C NMR data. Fortunately, compound 5 dissolved in trifluoroacetic acid (TFA), forming the salt 5H_2_^2+^ 2TFA^−^ (referred to as 5H_2_^2+^). X-ray quality single crystals were obtained from a hot PhCl/TFA solution that was left to slowly cool at room temperature over several days. The asymmetric unit cell was *P*2_1_/*n* monoclinic and revealed the formation of [5H_2_^2+^ 2TFA^−^]·2TFA. This structure represents the pyridinium dication 5H_2_^2+^ co-crystallised with two molecules of TFA and two TFA anions (Fig. 1). In addition, the C–O bond lengths (*d*_C–O_ = C24–O2 1.319(3) Å and C9–O1 1.326(3) Å) suggest that, in this protonated form, compound 5 exists in the enol tautomer instead as the keto tautomer, which was the case with product 3. TD-DFT calculations at the B3LYP/6-311G(d,p) level of theory confirmed this (ESI, S2.3†), as the calculated UV-vis absorption of the enol tautomer of the neutral molecule and the pyridinium form in the presence of coordinated anionic and neutral TFA molecules is in good agreement with the experimental UV-vis absorption in TFA as solvent (ESI, S2.3†). The crystal structure showed that the quinolinols deviate from the plane of the BTZ and the torsion angle between the planes defined by each quinolinol and the BTZ is 126.11(17)°. Support for this propeller-type geometry in solution came from the NOE 2D NMR spectrum (ESI, S3†) of product 5H_2_^2+^, which supported a nonplanar geometry, as there was no interaction between the BTZ and quinolinol protons, showing that the two quinolinol moieties must be nearly orthogonal with respect to each other, from a top-bottom perspective. Both neutral and protonated compounds were thermally stable up to at least 400 °C as revealed by TGA analysis (ESI, S2.1†).

### Photochemical studies

As mentioned above, the presence of the chromophore BTZ core in compound 5, prompted an investigation of its ability to act as a photosensitiser for reactive oxygen species (ROS) generation. More specifically, we decided to focus on the production of singlet oxygen (^1^O_2_), as it has a high synthetic utility^22^ and it is believed to be the main damaging agent in photodynamic therapy (PDT) applications.^23^ The formation of ascaridole *via* a Diels–Alder reaction between singlet oxygen (^1^O_2_) and α-terpinene was undertaken to evaluate the ability of compound 5 to act as a photosensitiser for the generation of ^1^O_2_.^15^ Owing to the low solubility of compound 5 (ESI, 2.7†), we carried out the reaction heterogeneously by using continuous-flow technology. This alternative enabling technology is an effective method for use with heterogeneous photosensitisers, and can offer better results compared to batch experiments, as it was recently demonstrated by our group.^24^ For this work, 1 mol% of compound 5 (0.0025 mmol) was mixed with 500 mg of silica (solid support). The mixture was then added into a short column reactor and was ready for use. To take full advantage of the flow instrumentation capabilities, we connected an O_2_ gas line to the set-up and the operating overall system pressure was set at 2 bar, allowing more O_2_ to be dissolved in solution.

**Fig. 2:**
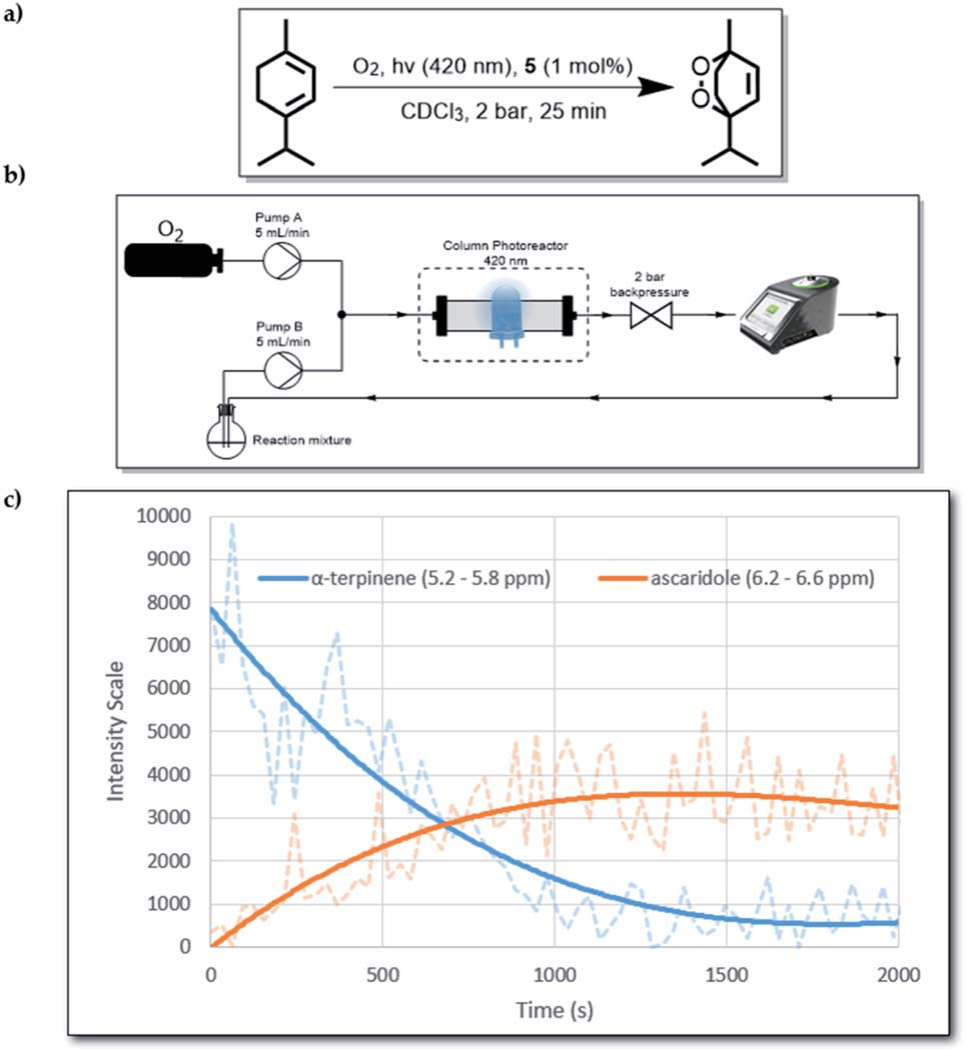
(a) The overall reaction from α-terpinene to ascaridole. (b) Schematic diagram of the flow set-up. (c) In-line ^1^H NMR data for the first run of the reaction.

Moreover, a 60 MHz Nanalysis-60e benchtop ^1^H NMR instrument was connected to the set-up, which allowed us to monitor the reaction progress in real time (Fig. 2). To make the ^1^H NMR data acquisition possible, CDCl_3_ was used as the solvent, which also offers significantly increased lifetime of ^1^O_2_ in solution compared to non-deuterated and non-chlorinated solvents.^25^ While compound 5 absorbs mostly in the long UV region of the spectrum (ESI, S1.4†), we used an LED emitting at 390–440 nm to test whether its absorbance at 400–420 nm was enough to produce its excited state. Gratifyingly, when the reaction was tested using the aforementioned parameters, in-line ^1^H NMR spectroscopy indicated that after 1500 s (25 min) the reaction was complete.

Since α-terpinene reacts selectively with ^1^O_2_,^18^ by calculating its conversion to ascaridole, the amount of generated ^1^O_2_ can be estimated. For the optimised reaction conditions above, this was found to be 0.54 mmol h^−1^ per mg of 5. This performance is comparable with previously described BTZ-based heterogeneous photocatalysts that were used under continuous flow conditions (Table 1). Even though its performance is not particularly outstanding, what makes compound 5 stand out is that it is the first example of a molecular based BTZ heterogeneous photocatalyst that has been used for ^1^O_2_ production. A comparison between the ^1^O_2_ productivities of compounds 5 and 5H_2_^2+^ was also made, to test if they behave differently under identical conditions, however there was negligible difference in their performance (ESI, S2.8†).

**Table 1:** ^1^O_2_ generation efficiencies of various BTZ-based heterogeneous photosensitisers under continuous flow conditions and visible light (420 nm) illumination

Entry	Photosensitiser characteristics	Reactor type	^1^O_2_ productivity^a^ (mmol h^−1^)
1 (ref. 16)	Conjugated microporous BTZ polymer	Coil	1.44
2 (ref. 18)	High internal phase emulsion BTZ polymer	Column	0.15
3 (ref. 15)	High internal phase emulsion BTZ polymer	Column	0.05
4 (ref. 15)	Polymer supported BTZ (microbeads)	Coil	4.04
5-Current work	Molecular BTZ, SiO_2_ supported	Column	0.54

a Per mg of photosensitiser.

To test the durability of compound 5 as a photosensitiser, we repeated the same reaction four more times without replacing the original packed column. By ^1^H NMR analysis, we observed that even after 5 cycles, there was quantitative consumption of α-terpinene (0.25 mmol per cycle) without any apparent photo-bleaching of 5, judging by its consistent performance. This behaviour highlights the excellent stability of 5 against ^1^O_2_. The graph (Fig. 2c) follows signal intensity for two specific spectral regions which correspond to α-terpinene and ascaridole. The actual real time data are represented by the faded dashed lines, which were used to generate a moving average trend line. The noise in the raw data is partly attributed to the flow of O_2_ bubbles through the bench-top ^1^H NMR instrument, which lowered the signal quality. Nevertheless, the signal was accurate enough to provide real-time qualitative information about the reaction progression. To confirm reaction completion, after each run the content of the collection flask was isolated by removing the solvent under reduced pressure and ^1^H NMR spectra using a 300 MHz instrument were obtained (ESI, S2.5†). It was also observed that when alumina was used instead of silica as a solid support, poor adsorption of compound 5 resulted in gradual leaching of solids into the reaction mixture (ESI, S2.5†).

To prove that the reaction was catalysed heterogeneously and not due to low concentrations of soluble 5, a hot suspension of compound 5 (1 mol%) in CDCl_3_ was stirred for 30 min, and the solids were then removed by filtration. To the resulting solution was added α-terpinene (0.25 mmol). After irradiating the solution, no ascaridole was detected, proving that luminogen 5 acts as a true molecular heterogeneous photocatalyst (ESI, S2.5†).

Finally, as we have shown that 5 can produce a stable excited triplet state for the subsequent type II energy transfer to occur, we wanted to assess whether it can also participate in type I photosensitised reactions, to produce other types of ROS, such superoxide (O_2_˙^−^). As such, we used compound 5 as a photoredox catalyst for the aerobic hydroxylation of phenylboronic acid to phenol. This reaction has been shown to be mediated by O_2_˙^−^,^26^ and when 1 mol% of 5 was used, there was 36% conversion to phenol, as measured by HPLC (ESI, S2.9†).

### Photophysical studies

**Fig. 3:**
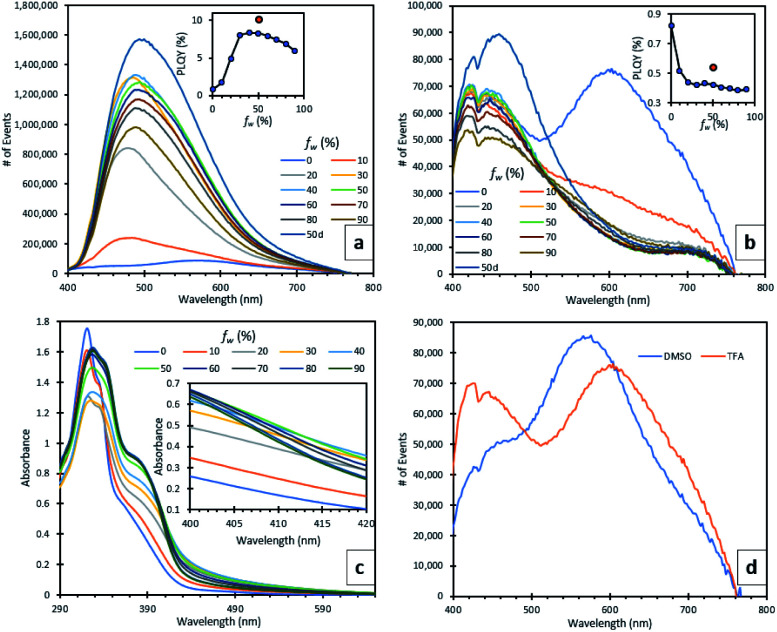
(a) DMSO/H_2_O series emission spectra. Inset shows relation between *f*_w_ and PLQY (%). (b) TFA/H_2_O series emission spectrum. Inset shows relation between *f*_w_ and PLQY (%). (c) DMSO/H_2_O series absorption spectrum. Inset shows an enlarged section between 400 nm and 420 nm. (d) Comparison of emissions between DMSO and TFA, while *f*_w_ was 0. *C* = 2.05 × 10^−4^ M. Excitation wavelength was 320 nm.

Due to the insolubility of compound 5 in organic solvents, its photoluminescence behaviour was only studied in the solid (semi-crystalline) state. A broad green emission at 512 nm was observed (ESI, S2.4†). The protonated TFA salt 5H_2_^2+^ was slightly more soluble in polar organic solvents such as DMSO and thus, it was used to make dilute solutions in different ratios of DMSO/water and TFA/water (water fraction, *f*_w_ ranged from 0 to 90%) and their fluorescence spectra were recorded (Fig. 3a and b). At 100% DMSO (Fig. 3d), the emission spectrum was weak, containing two peaks, a sharp one at 428 nm which can be attributed to the singlet excited state and a broad one at 446 nm. The rotatable, D–A bond between the quinolinol and BTZ moieties indicated that this broad peak may be the result of intramolecular charge transfer (ICT) or possibly twisted intramolecular charge transfer (TICT).^27^ TD-DFT calculations showed that in the molecular orbitals associated with the vertical excitations responsible for the absorption profile of 5H_2_^2+^ the HOMO to LUMO transition has significant charge transfer character from the quinolinol moieties to the BTZ core (ESI, S2.3†), which further strengthens the case of ICT emission. A third, redshifted broad peak at 574 nm is believed to originate from an excimer, which forms a broad UV-vis absorption band at 460 nm with increasing concentrations (ESI, S2.4†). When *f*_w_ was increased to 10% a fine precipitate started to appear with concomitant disappearance of the excimer peak, bathochromic and hyperchromic transformation of the ICT peak, and appearance of a new, broad peak, which continued to increase in intensity until *f*_w_ reached 40% (Fig. 3a). At this point, there was a 24-fold increase in emission intensity and 10-fold increase in photoluminescence quantum yield (PLQY), which reached 8.4%. The peak continued to redshift until 490 nm when *f*_w_ reached 50%. This can be attributed to ICT solvatochromism^28^ and shows that both AIE and ICT effects are active initially. Between *f*_w_ 50–90% the emission decreased, a rarely observed behaviour seen in other AIE systems which has been ascribed to changes in the crystallisation processes and available surface area of the nanoaggregates, as more water is added.^29^

The UV-vis absorption spectra of the DMSO/water series show two new shoulders appearing at 371 and 410 nm (Fig. 3c). The latter follows the same behaviour as the emission spectra, showing a maximum absorbance when *f*_w_ is 40% and decreasing thereafter. To verify the relationship of this absorption shoulder with the formation of nanoaggregates, we used varying excitation wavelengths spanning from 320 nm (*λ*_abs,max_ of 5H_2_^2+^) to 430 nm to monitor the AIE emission peak at 478 nm. When *λ*_exc_ was 410 nm, the *λ*_em_ intensity was maximised. When TFA was used instead of DMSO, no aggregation was observed until *f*_w_ reached 80%, confirmed by the corresponding UV-vis absorption spectra (ESI, S2.4†), showing a significant level-off tail above 400 nm, which is due to the light scattering caused by the nanoaggregate suspension.^3^ In spite of this, no AIE peak emerged, which can be explained by TFAs ability to form strong intermolecular H-bonds between the phenolic and pyridinium moieties of compound 5H_2_^2+^, as seen by the crystal structure. These H-bonds can in turn induce non-radiative pathways, by competing with the radiative ICT processes between the BTZ and quinolinol moieties.^30^ To further demonstrate this point, when D_2_O was used instead of H_2_O for the solutions with *f*_w_ of 50%, their fluorescence was enhanced, increasing their PLQYs for the DMSO and TFA solutions by 22 and 29%, respectively. A striking difference was seen in the TFA spectra, where the weaker ICT/AIE peak became more intense than the monomeric peak while also getting broader and bathochromically shifted to 462 nm (Fig. 3b). This effect is associated to the inferior quenching abilities of deuterium,^31^ and strengthens the case of H-bonding having an important role in the quenching of the AIE peak in the TFA solution. Finally, an aerated TFA/D_2_O (*f*_w_ = 50%) solution exhibited a weak emission at 1274 nm (ESI, S2.4†), caused by ^1^O_2_ phosphorescence, which directly supported its ability to generate ^1^O_2_, even in solution.^32^

## Conclusions

In summary, we have discovered a robust and easily accessible D–A–D chromophore with a twisted geometry. Its high insolubility allowed us to use it as a very effective molecular heterogeneous photosensitiser for ^1^O_2_ production under continuous flow conditions. In addition, we explored the photophysical properties of its TFA salt, which revealed a tuneable dual emission between a redshifted excimer and a combination of ICT/AIE which was controlled by adjusting the water fraction. When TFA was used as a solvent the AIE emission was turned off due to increased H-bonding. This study acts as a gateway for further investigation of this unique class of quinolinol-BTZ AIEgen triads, and we think the combination of their facile synthesis, tunability, heterogeneous ^1^O_2_ generating capabilities and potential antimicrobial properties make them attractive targets for future research.

## Author contributions

Synthesis, photochemical and photophysical investigations, E. B.; PLQY measurements and instrumentation access, C. J, J. M-H.; computational studies and SC-XRD data interpretation, M. K.; acquisition of MS data and SC-XRD data interpretation, G. O. L.; acquisition of SC-XRD data, A. K.; writing and draft presentation, E. B, M. K, G. O. L, P. A. K, and F. V.

## Conflicts of interest

There are no conflicts to declare.

## Supplementary Material

RA-011-D1RA06263H-s001

RA-011-D1RA06263H-s002
