# Supplementary material for: 8,8′-(Benzo[c][1,2,5]thiadiazole-4,7-diyl)bis(quinolin-4(1H)-one): a twisted photosensitizer with AIE properties
Source: RSC Adv. 2021 Sep 1;11(47):29102–7. doi: 10.1039/d1ra06263h (PMC9040641; doi:10.1039/d1ra06263h)
Supplement: RA-011-D1RA06263H-s001 [file RA-011-D1RA06263H-s001.pdf]

Electronic Supplementary Material (ESI) for RSC Advances.
This journal is © The Royal Society of Chemistry 2021

Supporting Information

8,8'-(Benzo[c][1,2,5]thiadiazole-4,7-diyl)bis(quinolin-4(1H)-one): A Twisted Photosensitizer with AIE Properties

Emmanouil Broumidis,[1] Callum M. S. Jones,[2] Maria Koyioni,[3] Andreas Kourtellaris,[3] Gareth O. Lloyd,[4] Jose Marques-Hueso,[2] Panayiotis A. Koutentis,*,[3] and Filipe Vilela*,[1]

1 *Institute of Chemical Sciences, School of Engineering & Physical Sciences, Heriot-Watt University, Edinburgh, EH14 4AS, United Kingdom*

2 *Institute of Sensors, Signals and Systems, School of Engineering & Physical Sciences, Heriot-Watt University, Edinburgh, EH14 4AS, United Kingdom*

3 *Department of Chemistry, University of Cyprus, P.O. Box 20537, 1678 Nicosia, Cyprus*

4 *Joseph Banks Laboratories, School of Chemistry, University of Lincoln, Brayford Pool, Lincoln, LN6 7TS, United Kingdom*

* *Correspondence: F.Vilela@hw.ac.uk; koutenti@ucy.ac.cy*

Table of Contents

S1 Experimental Section S3

S1.1 General methods and materials S3

S1.2 Preparation of 5-{[(2-Iodophenyl)amino]methylene}-2,2-dimethyl-1,3-dioxane-4,6-dione **1** S4

S1.3 Preparation of 8-Iodoquinolin-4(1*H*)-one **2** S4

S1.4 Preparation of 8,8'-(Benzo[*c*][1,2,5]thiadiazole-4,7-diyl)bis(quinolin-4-ol) **5** S4

S1.5 Preparation of **5**H_2^{2+} from **5** S5

S1.6 Heterogeneous flow mediated photocatalytic generation of singlet oxygen with in-line NMR analysis S6

S1.7 Photoluminescence quantum yield (PLQY) calculations S7

S2 Complementary data and discussion S8

S2.1 TGA curves of **5** and **5**H_2^{2+} S8

S2.2 Crystallographic data of compound **5**H_2^{2+} S9

S2.3 Computational data S11

S2.4 Additional photophysical data S19

S2.5 Continuous flow set-up and cycle data S23

S2.6 X-Ray powder diffraction (XRD) of **5** S27

S2.7 Solubility of **5** in various organic solvents S28

S2.8 $^1O_2$ generation efficiencies of **5** and **5**H_2^{2+} under indentical conditions S28

S2.9 Aerobic oxidative hydroxylation of phenylboronic acid (HPLC data) S29

S3 NMR spectra of compounds **1**, **2**, **5** and **5**H_2^{2+} S30

S4 MS spectra of compounds **5** and **5**H_2^{2+} S46

S5 References S48

S1 Experimental Section

S1.1 General methods and materials

All chemicals were commercially available except those whose synthesis is herein described. Anhydrous $MgSO_4$ was used for drying organic extracts and all volatiles were removed under reduced pressure. All reaction mixtures and column eluents were monitored by TLC using commercial glass backed thin layer chromatography (TLC) plates (Merck Kieselgel 60 F_{254}). The plates were observed under UV light at 254 and 365 nm. The technique of flash chromatography was used throughout for all non-TLC scale chromatographic separations using Merck Silica Gel 60 (less than 0.063 mm). Melting points were determined using a Stuart SMP10 digital melting point apparatus. Small scale (μL) liquid handling measurements were made using variable volume (1.00–5000.00 μL) single channel Gilson PIPETMAN precision micropipettes. Solvents used for recrystallisation are indicated after the melting point. IR spectra were recorded on a Thermo Scientific Nicolet iS5 FTIR spectrometer with iD5 ATR accessory and broad, strong, medium and weak peaks are represented by b, s, m and w, respectively. ^{1}H and ^{13}C NMR spectra were recorded on a Bruker AVANCE III HD machine (at 400 and 100 MHz, respectively). An AVANCE III 300 MHz NMR Spectrometer was also used for reaction monitoring. The benchtop NMR that was used for flow reaction monitoring was a NANALYSIS corp. NMRReady 60 Benchtop ^{1}H NMR. Chemical shifts (δ) are expressed in ppm and coupling constants *J* are given in Hz. Data are represented as follows: chemical shift, multiplicity (s = singlet, d = doublet, t = triplet, q = quartet, m = multiplet and/or multiple resonances, br s = broad singlet). Deuterated solvents were used for homonuclear lock and the signals are referenced to the deuterated solvent peaks. For the acquisition of mass spectra the samples were prepared as detailed below and analysed by positive ion nanoelectrospray (nES) using a Thermo Scientific™ LTQ Orbitrap XL™ ETD Hybrid Ion Trap-Orbitrap Mass Spectrometer. The absolute PLQY of of each sample was measured using a calibrated spectrofluorometer (Edinburgh Instruments, FLS920), equipped with an integrating sphere (Jobin-Yvon). Flow reactions were carried out with a commercial E-series Photochem reactor by Vapourtec Ltd, with two V-3 peristaltic pumps and using an LED module emitting at 390-440 nm with 7.44 W light output (EPILED, Future Eden Ltd.). For the collection of LC-MS data a Shimadzu LC-2040C 3D Plus instrument was used.

S1.2 Preparation of 5-{[(2-Iodophenyl)amino]methylene}-2,2-dimethyl-1,3-dioxane-4,6-dione **1**

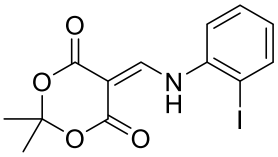

To a stirred solution of 2-iodoaniline (3.00 g, 13.6 mmol), in anhydrous acetonitrile (50 mL) at *ca.* 20 °C was added in one portion Meldrum's acid (2.15 g, 15.0 mmol), followed by triethyl orthoformate (2.41 g, 16.3 mmol). The resulting mixture was then heated to *ca.* 95 °C (reflux) for 2 h until the 2-iodoaniline was consumed (by TLC). The resulting mixture was then allowed to cool to *ca.* 20 °C, evaporated to dryness under vacuum, and the residue recrystallised by hot ethanol (EtOH) to afford the title compound **1** (4.01 g, 78%) as orange cubes, mp (hot-stage) 177-178 °C (EtOH) [literature value: 176.1-177.9 °C (EtOH)][1]; R_f 0.44 (*n*-hexane/EtOAc, 60:40); ν_{max}/cm^{-1} 3163w (aryl C-H), 2995w (alkyl C-H), 1726m and 1680s (C=O), 1601w, 1582m, 1568m, 1493w, 1433w, 1389m, 1377m, 1319s, 1300m, 1269s, 1227w, 1200s, 1144w, 1119w, 1016m, 993w, 984m, 930m, 889w, 845w, 806m, 779m, 746s, 723m; δ_H (400 MHz, DMSO-d_6) 11.37 (1H, d, J = 14.0 Hz, N*H*), 8.65 (1H, d, J = 14.0 Hz, =C*H*), 7.93 (1H, dd, J = 7.6 Hz, 1.2 Hz, Ar *H*), 7.73 (1H, dd, J = 8.0 Hz, 0.8 Hz, Ar *H*), 7.46-7.50 (1H, m, Ar *H*), 7.05 (1H, ddd, J = 7.8 Hz, 7.8 Hz, 1.6 Hz, Ar *H*), 1.69 (6H, s, CH_3); δ_C (100 MHz, DMSO-d_6) 164.6, 162.3, 153.5, 139.6, 139.2, 129.9, 128.0, 118.6, 104.6, 90.7 87.7, 26.6; identical to an authentic sample.[1]

S1.3 Preparation of 8-Iodoquinolin-4(1*H*)-one **2**

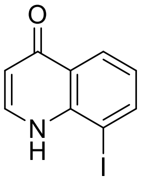

To stirred diphenyl ether (5 mL) preheated to *ca.* 220 °C (Wood's metal bath), was added portion wise over a 4 min period 5-{[(2-iodophenyl)amino]methylene}-2,2-dimethyl-1,3-dioxane-4,6-dione **1** (1.00 g, 2.7 mmol) and the mixture was heated at *ca.* 220 °C for another 5 min until the starting material was consumed (by TLC). The resulting mixture was then allowed to cool to *ca.* 20 °C, diluted (*n*-hexane, 15 mL) and the insoluble precipitate was isolated by filtration and recrystallised by hot toluene (PhMe) to afford the title compound **2** (679 mg, 93%) as beige-colored cubes, mp (hot-stage) 76-77 °C (PhMe) [literature value: 75.3-76.3 °C (PhMe)][1]; R_f 0.41 (THF/EtOAc, 50:50); ν_{max}/cm^{-1} 3090 brw (N-H), 1618s (C=O), 1585s, 1543s, 1504s, 1431m, 1325m, 1300w, 1240m, 1215w, 1188s, 1099w, 1074w, 1051m, 889w, 866w, 835w, 793s, 775m, 746s; δ_H (400 MHz, DMSO-d_6) 10.72 (1H, br s, N*H*), 8.18 (1H, dd, J = 7.6 Hz, 1.2 Hz, Ar *H*), 8.11 (1H, dd, J = 8.0 Hz, 1.2 Hz, Ar *H*), 7.88 (1H, d, J = 6.8 Hz, Ar *H*) 7.11 (1H, dd, J = 8.0 Hz, 8.0 Hz, Ar *H*), 6.13 (1H, d, J = 6.0 Hz, Ar *H*); δ_C (100 MHz, DMSO-d_6) 176.7, 142.2, 140.7, 140.4, 126.6, 125.6, 124.7, 109.0, 87.4; identical to an authentic sample.[1]

S1.4 Preparation of 8,8'-(Benzo[*c*][1,2,5]thiadiazole-4,7-diyl)bis(quinolin-4-ol) **5**

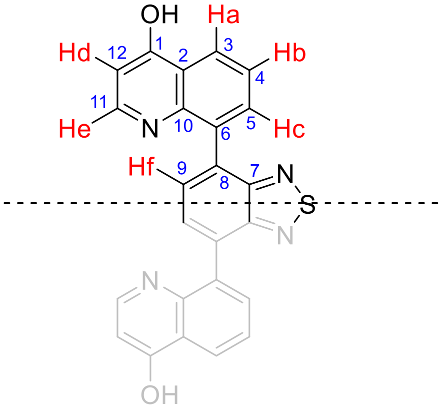

To a stirred solution of 8-iodoquinolin-4(1*H*)-one **2** (100.0 mg, 0.368 mmol) in dioxane/H_2O (75:25) (1 mL) at *ca.* 20 °C was added in sequence powdered K_2CO_3 (101.6 mg, 0.720 mmol), followed by 4,7-bis(4,4,5,5-tetramethyl-1,3,2-dioxaborolan-2-yl)benzo[*c*][1,2,5]thiadiazole **4** (71.4 mg, 0.184 mmol), and then Pd(dppf)$Cl_2\cdot CH_2Cl_2$ (15.0 mg, 5 mol %). The reaction mixture was then immersed into a preheated oil bath and heated at *ca.* 89 °C (reflux) for 5 min until the iodoquinolinone **2** was consumed (by TLC, THF/EtOAc, 50:50). The reaction mixture was then allowed to cool to *ca.* 20 °C leading to the formation of a powdery yellow precipitate, which was isolated

by filtration, washed thoroughly using THF (2 × 5 mL), $CHCl_3$ (2 x 5 mL) and EtOH (2 × 5 mL), and then dried under a stream of air, to afford the title compound **5** (75.2 mg, 97%) as a fine yellow powder, mp (TGA) > 400 °C (decomp.); λ_{max}(DMSO)/nm 306 inf (log ε 3.42), 317 (3.48), 325 (3.35); ν_{max}/cm^{-1} 3166brw (C-H), 1619s, 1617s, 1567s, 1506s, 1433m, 1403w, 1335m, 1310w, 1284w, 1247w, 1191s, 1146w, 1122m, 1066m, 979w, 975m, 901w, 880m, 860w, 849m, 810m, 795m, 756s; δ_H (400 MHz, DMSO-d_6) 8.28 (2H, dd, J = 8.0 Hz, 1.2 Hz, Ar H_a), 7.94 (2H, s, Ar H_f), 7.75 (2H, dd, J = 7.2 Hz, 1.2 Hz, Ar H_e), 7.65 (2H, d, J = 7.2 Hz, Ar H_c), 7.47 (2H, dd, J = 7.6 Hz, 7.6 Hz, Ar H_b), 6.07 (2H, d, J = 7.2 Hz, Ar H_d); m/z (ESI+): calculated for $[M+H]^+$ 423.0910, found 423.0905.

S1.5 Preparation of **5**H_2^{2+} from **5**

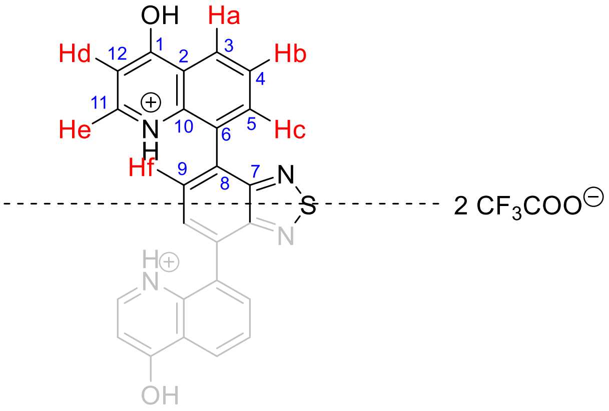

To a 5 mL glass vial was added compound **5** (20.0 mg, 0.047 mmol) in 1 mL of TFA. The reaction mixture was then allowed to stir at *ca.* 20 °C for 10 minutes at which point all of the solid was dissolved. The contents of the vial were then transferred into a 50 mL conical flask containing 10 mL of distilled water, leading to the formation of a powdery dark yellow precipitate, which was isolated by filtration, washed thoroughly using cold distilled water (2 × 5 mL) and acetone (2 × 5 mL), and then dried under a stream of air, to afford the title compound **5**H_2^{2+} (25.0 mg, 82%) as a fine yellow powder. Crystals of **5**H_2^{2+} were grown by dissolving a small amount (~5 mg) in a 1:1 mixture (1 mL) of hot chlorobenzene (PhCl) and TFA. mp (TGA) > 400 °C (decomp.) (TFA/PhCl); found: C, 51.30; H, 2.42; N, 8.43. $C_{28}H_{16}F_6N_4O_6S$ requires: C, 51.70; H, 2.48; N, 8.61%; λ_{max}(TFA)/nm 306 inf (log ε 3.42), 317 (3.48), 325 (3.35); ν_{max}/cm^{-1} 3166brw (C-H), 1619s, 1617s, 1567s, 1506s, 1433m, 1403w, 1335m, 1310w, 1284w, 1247w, 1191s, 1146w, 1122m, 1066m, 979w, 975m, 901w, 880m, 860w, 849m, 810m, 795m, 756s; δ_H (400 MHz, TFA-*d*) 8.73 (2H, dd, J = 8.4 Hz, 1.2 Hz, Ar H_a), 8.57 (2H, d, J = 6.8 Hz, Ar H_e), 8.25 (2H, dd, J = 7.2 Hz, 1.2 Hz, Ar H_c) 8.15 (2H, s, Ar H_f), 7.04 (2H, dd, J = 7.2 Hz, 1.2 Hz, Ar H_b), 7.39 (2H, d, J = 6.8 Hz, Ar H_d); δ_C (100 MHz, TFA-*d*) 172.8 (1), 155.6 (7), 146.7 (11), 140.0 (10), 139.3 (6), 134.8 (9), 131.3 (8), 130.8 (4), 129.7 (5), 127.4 (3), 123.0 (2), 107.7 (12); δ_F (376 MHz, DMSO-d_6) -74.49.; m/z (ESI-): calculated for $[M\text{-}TFA\text{-}H]^-$ 535.0693, found 535.0665.

S1.6 Heterogeneous flow mediated photocatalytic generation of singlet oxygen with in-line NMR analysis

The procedure for the heterogeneous flow photosensitisation of **5** to produce singlet oxygen was adapted from Thomson *et. al*,[2] and was carried out as follows:

To a transparent borosilicate glass column fixed bed reactor (6.6 mm ID, 100 mm length) was added a mixture of photocatalyst **5** (1.1 mg, 0.0025 mmol, 1 mol%) and 500 mg chromatography grade (60A, 40 – 63 µm particle size) SiO_2. The dry-packed mixture was then attached to the Vapourtec flow machine and it was primed with $CDCl_3$ (10 mL) for 5 min by flowing solvent through the column at 5 mL/min. To a dry 20 mL round bottom flask loaded with a magnetic stirrer bar, was added *α*-terpinene (34.1 mg, 40.1 µL, 0.25 mmol) and $CDCl_3$ (5 mL) before sealing with a septum. The flask was connected to the Vapourtec flow machine and then covered to prevent photosensitisation events from ambient light. The reaction solution was pumped at 5 mL/min using peristaltic pumps (Vapourtec V-3) to a T-junction where it was mixed with O_2 gas from a cylinder which was being pumped concurrently at 5 mL/min by a second peristaltic pump. The heterogeneous liquid-gas mixture formed a slug flow at the T-junction and was pumped through the fixed bed column reactor placed in a reflective housing, irradiated by an LED array (390 - 440 nm, 7.44 W). After exiting the photoreactor, the slug flow was then passed through a back pressure regulator which kept the pressure at 2 bar before passing through a 60 MHz Nanalysis-60e benchtop ^{1}H NMR instrument which monitored the reaction progression in real time. Following that, the slug flow was returned to the initial reaction flask to be continuously cycled for 25 min. The catalyst bed was permitted to form a 'mixed bed' regime (hybrid between a packed bed and fluidised bed) to prevent pressure build up.

After the reaction was complete, as indicated by the bench-top NMR, the resulting solution was evaporated in vacuo to afford an oily residue. ^{1}H NMR (300 MHz) analysis of the residue revealed the complete disappearance of the α-terpinene alkene protons (δ_H 5.6–5.7 ppm) and the appearance of the corresponding ascaridole alkene protons (δ_H 6.4–6.6 ppm), confirming the bench-top NMR data and the complete transformation of the starting material.

For the cycling experiments, the loaded packed bed reactor was left in place, 15 mL of fresh $CDCl_3$ was flowed through the aforementioned set-up and it was then discarded. A new cycle could then be initiated by following the protocol above.

S1.7 Photoluminescence quantum yield (PLQY) calculations

The FLS920 spectrofluorometer was equipped with an extended red-sensitive single-photon counting photon multiplier (Hamamatsu, R2658P, 200-1010 nm), which was used to measure all spectra. A Xenon lamp, centered to 320 nm, was used as the excitation source.

The PLQY is defined by equation 1.[3] It is determined by dividing the number of photons emitted (L_{sample}), by the number of photons absorbed. The later is calculated by measuring the intensity of excitation light ($E_{reference}$) and subtracting it from the intensity of excitation light not absorbed by the sample (E_{sample}).

$$\mathrm{PLQY} = \frac{\#\ \text{photons emitted}}{\#\ \text{photons absorbed}} = \frac{\mathrm{L_{sample}}}{\mathrm{E_{reference}} - \mathrm{E_{sample}}} \qquad \text{Eq. 1}$$

L_{sample} = emission intensity

$E_{reference}$ = intensity of excitation light

E_{sample} = intensity of excitation light not absorbed by the sample

S2 Complementary data and discussion

S2.1 TGA curves of **5** and **5**H_2^{2+}

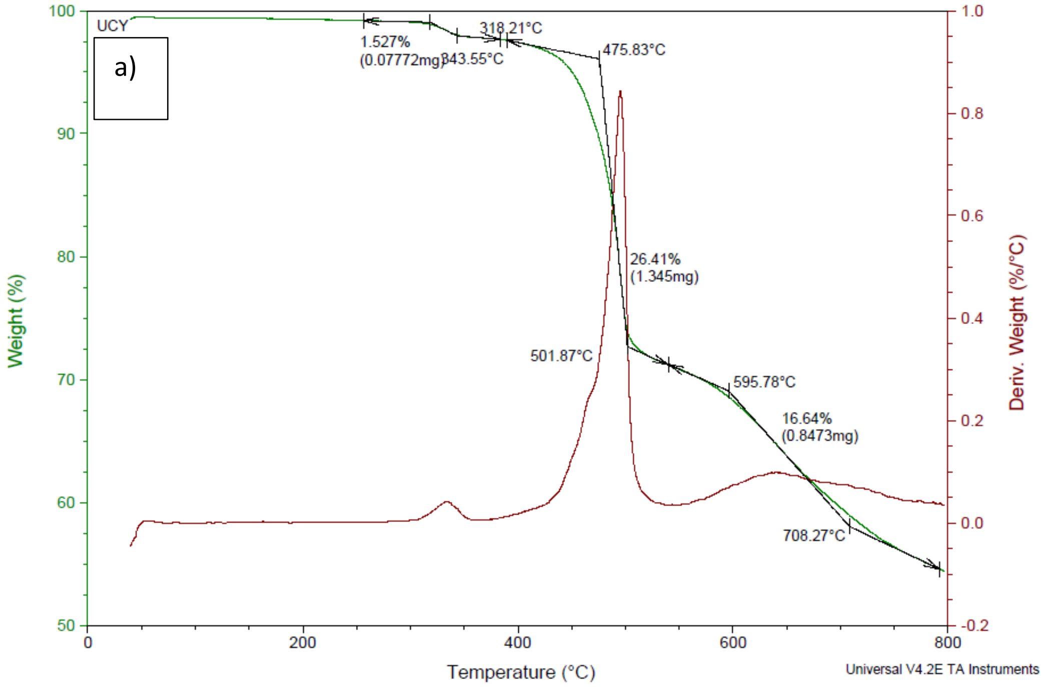

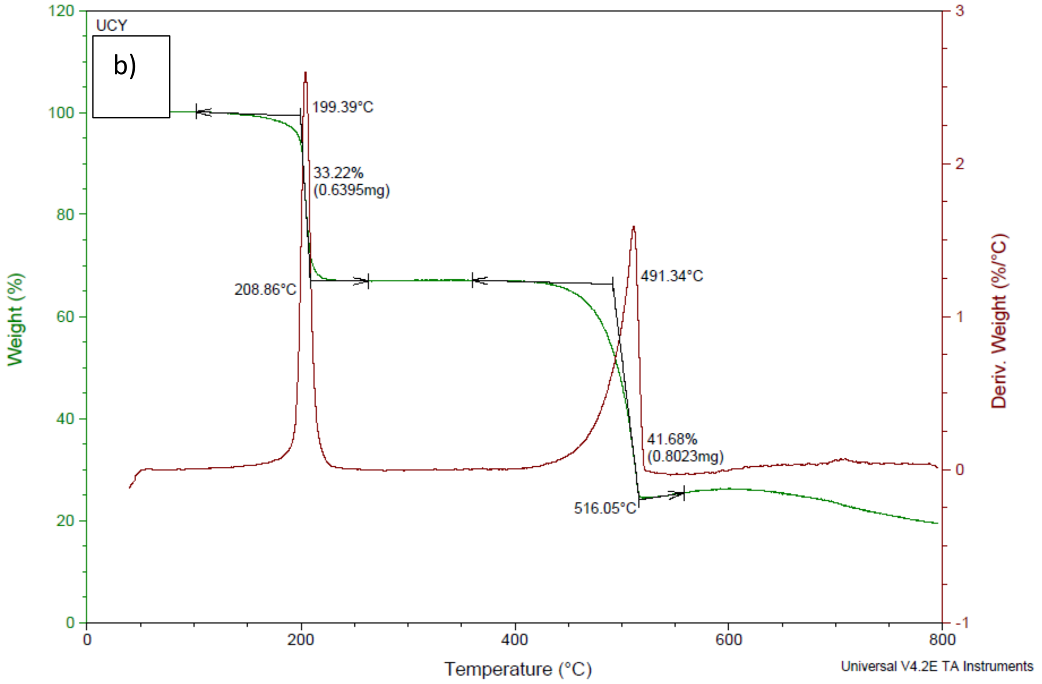

Figure S1. *a) TGA thermogram of freebase compound **5** has a very small % mass loss at around 320 °C which may be associated with a small impurity that is present in the sample. b) TGA thermogram of **5**H_2^{2+} in which the first % mass loss corresponds to the loss of the two TFA molecules.*

S2.2 Crystallographic data of compound **5**H_2^{2+}

The structure has been deposited with the CCDC with a Deposition Number 2086024.

Crystal data for [**5**H_2^{2+} $2TFA^-]\cdot 2TFA$**:** $[C_{24}H_{16}N_4O_2S^{2+}\ 2(C_2F_3O_2^-)].2C_2F_3O_2H$, M = 878.56, yellow plates, 0.18 x 0.04 x 0.03 mm^3, monoclinic, space group $P2_1/n$ (No. 14), a = 8.14670(10), b = 21.3962(3), c = 20.1733(3) Å, β = 90.509(2)°, V = 3516.24(8) $Å^3$, Z = 4, D_c = 1.660 g cm^{-3}, F_{000} = 1768, SuperNova, Dual, Cu at home/near, Atlas, MoK_α radiation, λ = 0.71065 Å, T = 100(2) K, $2\theta_{max}$ = 51.9°, 12906 reflections collected, 6738 unique (R_{int} = 0.0269). Final $GooF$ = 1.035, R_1 = 0.0513, wR_2 = 0.1380, R indices based on 5767 reflections with $I > 2\alpha(I)$ (refinement on F^2), 660 parameters, 0 restraints. Lp and absorption corrections applied, μ = 0.220 mm^{-1}.

Comparison of the calculated structures with the structure determined from the TFA- crystal of **5** indicates that the match in conformation and geometry is high. It appears structurally there is little difference in the protonation of **5** on distances or angles. This is highlighted by the low RMSD and maximum deviation numbers when comparing the molecular geometries (Table S1 and Figure S2).

Table S1. Comparison of the geometry parameters from the X-ray structure and the optimised structures in the gas phase at the RB3LYP/6-311G(d,p) level of theory.

**Structures compared**	RMSD (Å)	Maximum deviation (Å)
Crystal:[**5**H_2^{2+} $2TFA^-]\cdot 2TFA$	0.1947	0.3532
Crystal:[**5**H_2^{2+} $4TFA^-]\cdot 4TFA$	0.1775	0.3247
Crystal:**5**	0.0605	0.1507
[**5**H_2^{2+} $4TFA^-]\cdot 4TFA$:**5**	0.1876	0.3668

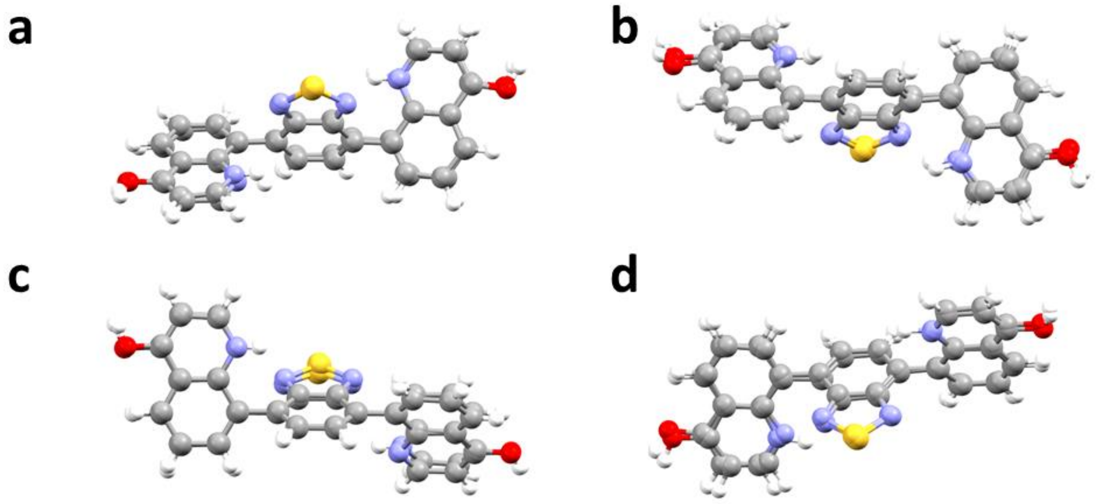

Figure S2. *Overlay of the crystal structure with the computed structures **5**, [**5**H_2^{2+} 4TFA-].4TFA and [**5**H_2^{2+} $2TFA^-]\cdot 2TFA$. a) Crystal:[**5**H_2^{2+} $2TFA^-]\cdot 2TFA$, b) Crystal:[**5**H_2^{2+} $4TFA^-]\cdot 4TFA$, c) Crystal:**5**, d) [**5**H_2^{2+} $4TFA^-]\cdot 4TFA$:**5**. Molcules in ball-and-stick representation and only molecules of **5** shown for clarity.*

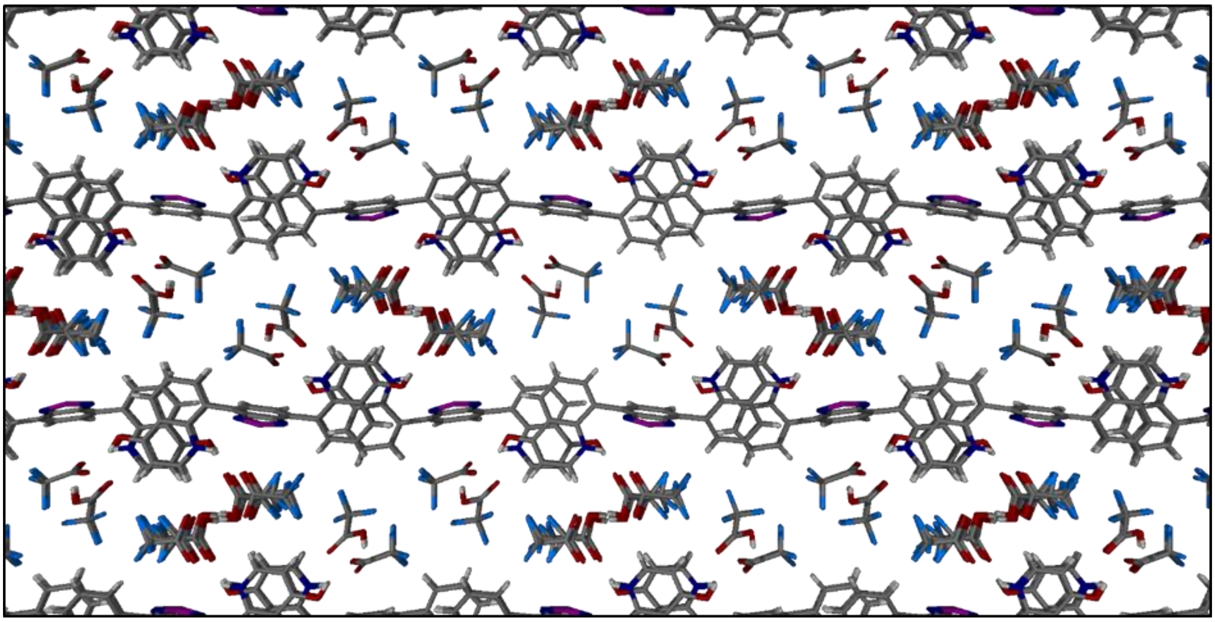

Figure S3. *Crystal packing structure of* ***5***H_2^{2+} *with the molecules shown as capped stick representations and viewed down the a axis. Disorder of one of the hydrogen bonded* CF_3COO^- CF_3COOH *pairs shown. π -π stacking of* ***5*** *results in layers of* ***5*** *and layers of* CF_3COO^- CF_3COOH*. There is extensive hydrogen bonding between the O–H and N–H groups of* ***5*** *and the donors and acceptors of the* CF_3COO^- *and* CF_3COOH*.*

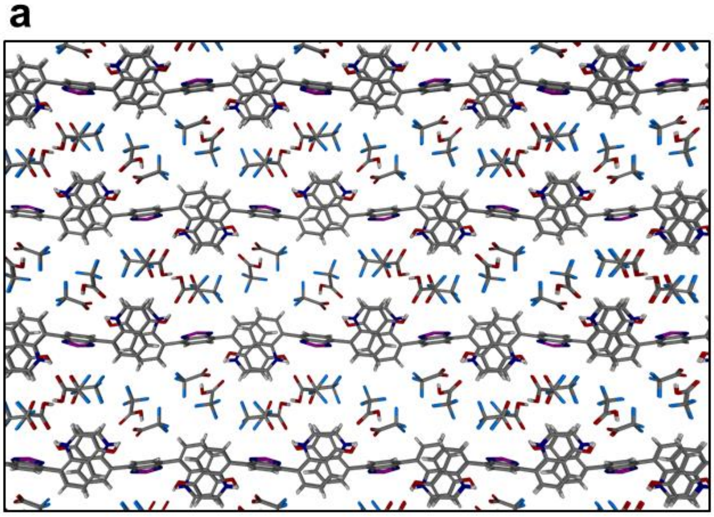

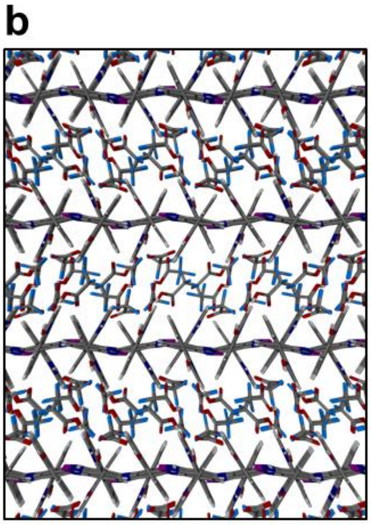

Figure S4. *Crystal packing structure of* ***5***H_2^{2+} *with the molecules shown as capped stick representation. a) Structure as viewed down the a axis. b) Structure as viewed down the c axis. Disorder not shown for clarity.*

S2.3 Computational data

The geometries of the molecule **5** in neutral and protonated form were fully optimized at the DFT RB3LYP/6-311G(d,p) level of theory and analytical second derivatives were computed using vibrational analysis to confirm each stationary point to be a minimum by yielding zero imaginary frequencies. TD-DFT calculations were performed also at the RB3LYP/6-311G(d,p) level of theory to obtain the vertical excitation energies. All the above computations were performed using the Gaussian 03 suite of programs.[5]

X-ray crystallography showed that molecule **5** exists in the solid state as the phenolic prototautomeric form **5''** cocrystallised with molecules of TFA that participate via various H-bonding interactions (Figure S5). In the absence of TFA or in the gas or solution phase the structure of compound **5** can differ. As two possible symmetrical prototautomers **5'** and **5''** exist, and one unsymmetrical, **5'''**, all were optimised at the RB3LYP/6-311G(d,p) level of theory in gas phase. The quinolone tautomer **5'** was computed to be 42.3 kJ·mol^{-1} more stable than prototautomer **5''** and 14.6 kJ·mol^{-1} more stable than **5'''**.

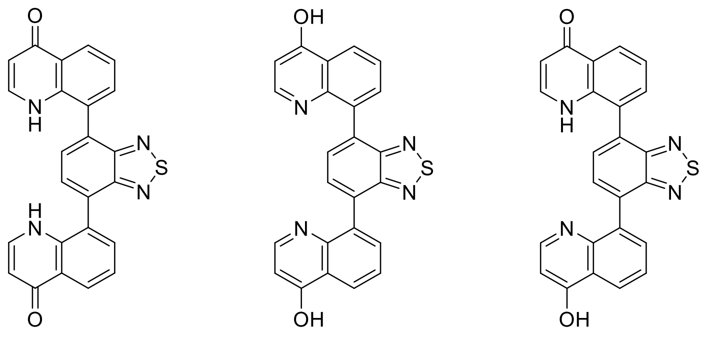

5' **5''** **5'''**

Figure S5. *Structures of the three possible prototautomers of compound **5**.*

TD-DFT calculations on the optimized structures of tautomers **5'**, **5''** and **5'''**, revealed a significant blue shift for the longest wavelength absorption of tautomer **5''** compared to **5'** and **5'''**, *ca.* 140 and 132 nm, respectively, with the absorption of the former being in best agreement with the experimental absorption spectra (Figure S6).

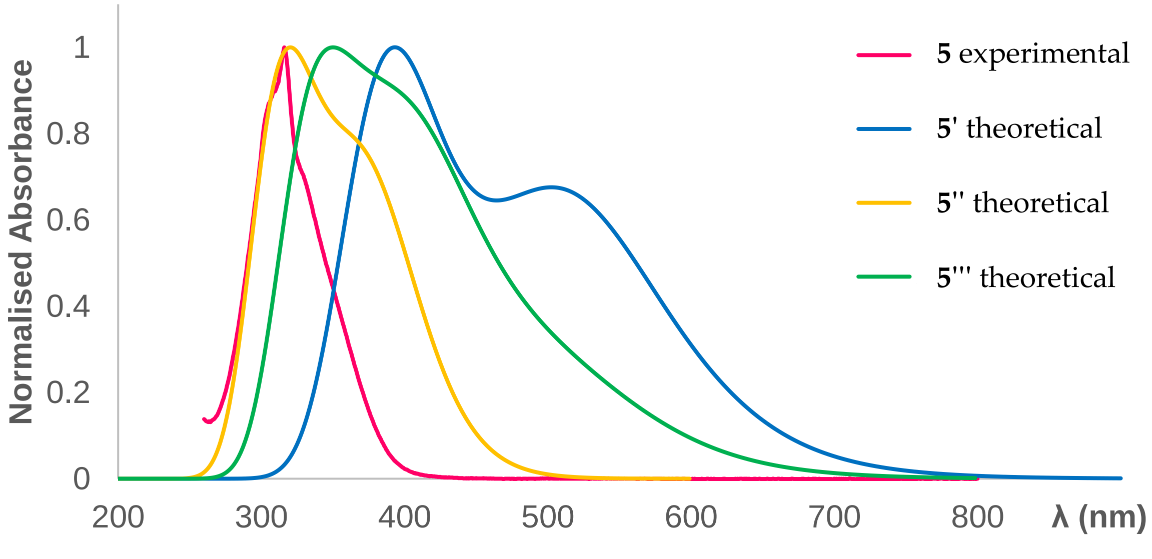

Figure S6. *Comparison of the experimental absorption spectrum for compound **5** and the theoretical absorption spectra for the prototautomers **5'**, **5''** and **5'''**.*

The main vertical excitations for molecule **5''** were calculated using TD-DFT at the B3LYP/6-311G(d,p) level of theory (Table S2), which indicated that the longest wavelength absorption corresponds to a HOMO → LUMO transition; observed as a shoulder in the theoretical and the experimental spectrum, blue shifted in the latter by *ca.* 51 nm). The two other absorptions observed theoretically at 322 and 312 nm, have a main contribution from a HOMO-2 → LUMO and a HOMO → LUMO+1 transition, respectively.

Table S2. Singlet excited states for **5''** from TD-DFT data at the B3LYP/6-311G(d,p) level of theory

excited state	transition (contribution)	energy (eV)	λ (nm)	osc. strength
**S1**	HOMO-2 → LUMO (2%)<br>**HOMO → LUMO (91%)**	**3.2951**	**376**	**0.1525**
S2	HOMO-1 → LUMO (98%)	3.5507	349	0.0007
**S3**	**HOMO-2 → LUMO (84%)**<br>HOMO → LUMO+1 (4%)	**3.8447**	**322**	**0.0955**
**S4**	HOMO-2 → LUMO (3%)<br>HOMO-2 → LUMO+1 (6%)<br>**HOMO → LUMO+1 (85%)**	**3.9741**	**312**	**0.1171**

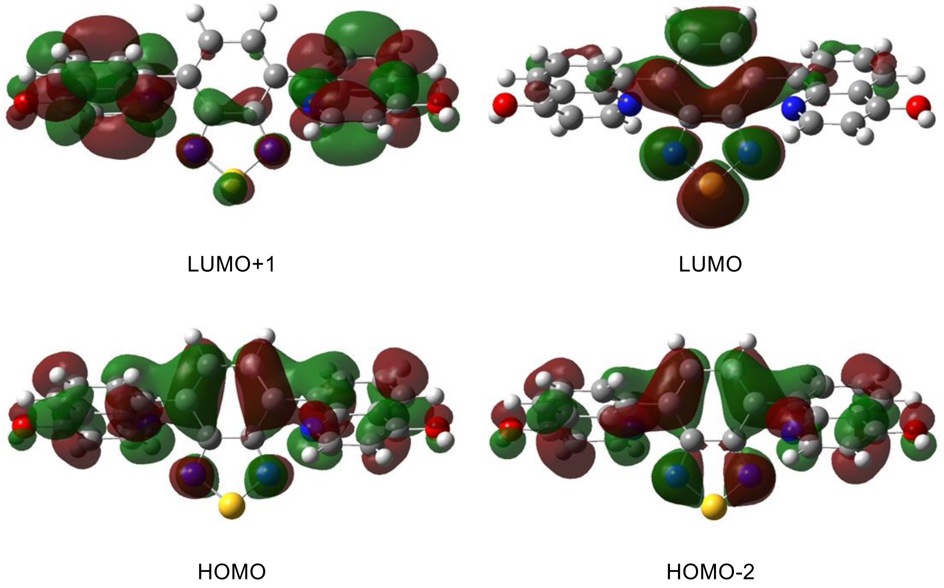

Figure S7. *The molecular orbitals associated with the vertical excitations responsible for the absorption profile of tautomer* ***5''*** *revealed that the HOMO → LUMO transition has significant charge transfer character from the quinolinol moieties to the benzothiadiazole core.*

As supported by X-ray crystallography, in TFA we expect the quinolinols to be in the protonated form, and as such the dication in the presence and absence of coordinated TFA molecules were studied computationally. In the absence of explicitly added TFA molecules in the cationic form the absorption is significantly red shifted owing to an intramolecular hydrogen bond between the NH of the

protonated quinolinol and the nitrogen of the thiadiazole ring. This leads to increased planarity of the system, and, as such, the absorption was shifted to longer wavelengths presumably owing to greater conjugation (Figure S8).

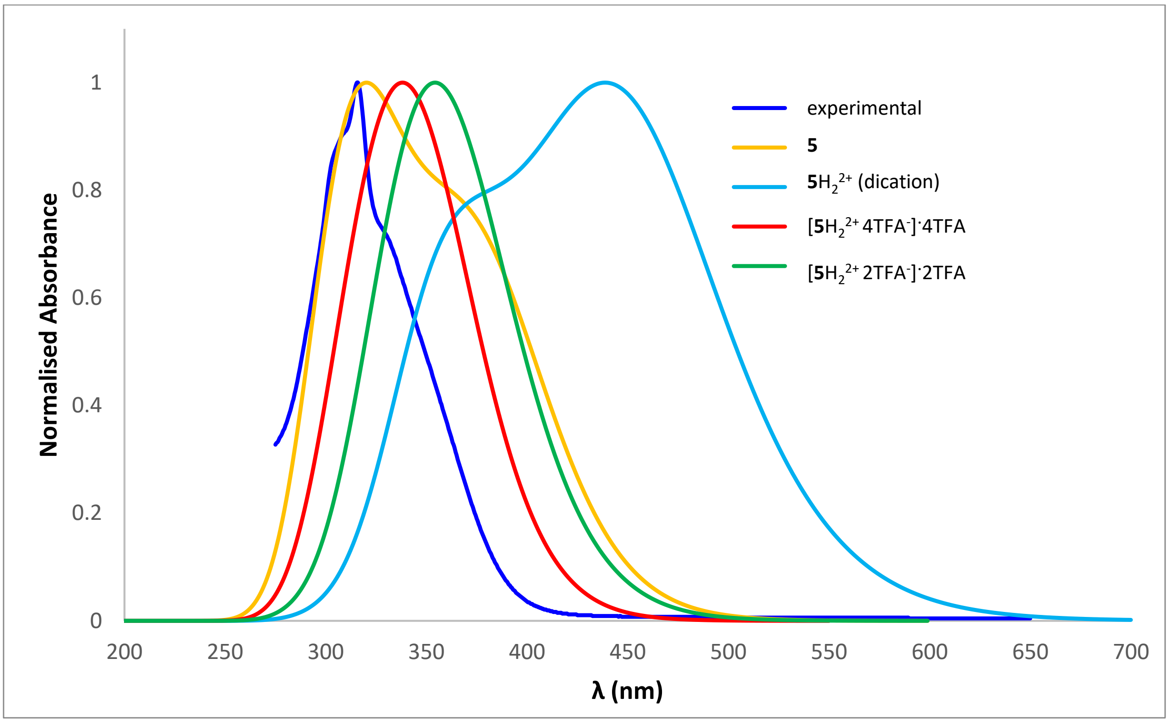

Figure S8. *Comparison of the experimental absorption spectrum for compound **5** and the theoretical absorption spectra for the neutral and dication species of **5**.*

The UV-vis absorption of the neutral molecule and the pyridinium form in the presence of coordinated anionic and neutral TFA molecules, however, is in good agreement with the experimental UV-vis absorption in TFA as solvent. The more TFA molecules incorporated into the calculation, the closer is the absorption to the experimental data in TFA.

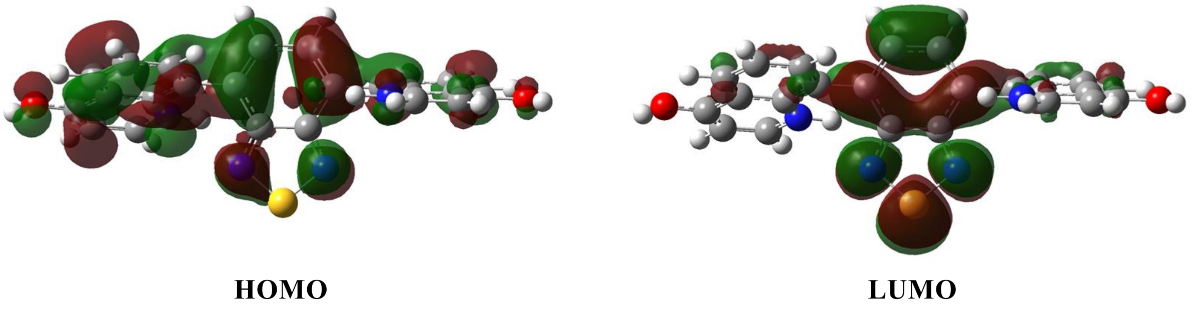

Figure S9. *HOMO and LUMO representations of the optimized structure of molecule **5** in the presence of eight TFA molecules (4 neutral and 4 anions, total charge -2). TFA molecules are omitted for clarity.*

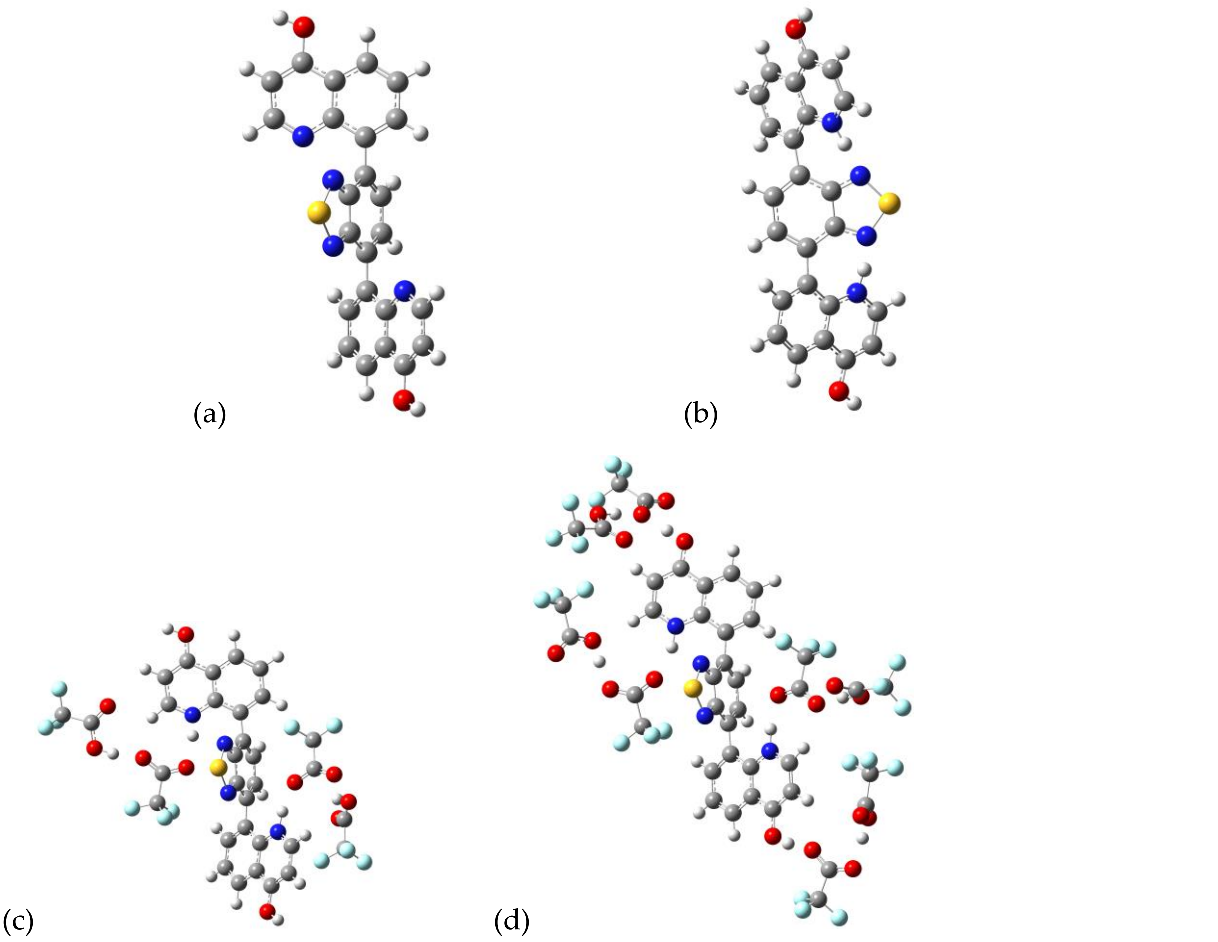

Figure S10. *Optimized structures of molecule* ***5*** *in (a) neutral form; (b) pyridinium form without counterion; (c) pyridinium form with two trifluoroacetates and two TFA; (d) pyridinium form with four trifluoroacetates and four TFA molecules.*

Atomic Cartesian Coordinates

8,8'-(Benzo[*c*][1,2,5]thiadiazole-4,7-diyl)bis(quinolin-4(1*H*)-one) (5')

Atom	Coordinates (Å)		
	x	y	z
C	-0.709799	1.416556	-0.155216
C	0.709800	1.416557	0.155212
C	1.434817	0.187251	0.314698
C	0.695079	-0.965350	0.155683
C	-0.695078	-0.965350	-0.155673
C	-1.434816	0.187249	-0.314696
H	1.181986	-1.924867	0.291713
H	-1.181985	-1.924869	-0.291699
N	-1.224708	2.644304	-0.265066
N	1.224709	2.644306	0.265052
S	-0.000001	3.696067	-0.000002
C	2.878926	0.175192	0.658756
C	3.823222	-0.557285	-0.107696
C	3.341266	0.880665	1.766665
C	5.179233	-0.599078	0.276486
C	4.684557	0.844111	2.152379
H	2.633507	1.460253	2.345747
C	5.589974	0.103289	1.416158
H	5.006897	1.395264	3.027652
H	6.638348	0.043905	1.682107
C	-2.878924	0.175188	-0.658756
C	-3.823222	-0.557284	0.107698
C	-3.341262	0.880654	-1.766671
C	-5.179232	-0.599079	-0.276486
C	-4.684552	0.844098	-2.152388
H	-2.633501	1.460238	-2.345755
C	-5.589971	0.103281	-1.416164
H	-5.006890	1.395245	-3.027664
H	-6.638345	0.043895	-1.682115
C	-6.184984	-1.367033	0.511349
C	6.184983	-1.367037	-0.511347
C	-5.652408	-2.021790	1.697310

C	-4.342803	-1.920073	2.024877	O	-7.363332	-1.426497	0.174998
C	4.342798	-1.920087	-2.024866	O	7.363332	-1.426499	-0.174998
C	5.652404	-2.021802	-1.697302	N	-3.446093	-1.227226	1.260314
H	-6.333597	-2.587205	2.318407	N	3.446090	-1.227235	-1.260306
H	-3.926426	-2.385439	2.910204	H	2.493561	-1.136487	-1.576551
H	3.926419	-2.385459	-2.910190	H	-2.493565	-1.136475	1.576561
H	6.333591	-2.587221	-2.318397				

8,8'-(Benzo[*c*][1,2,5]thiadiazole-4,7-diyl)bis(quinolin-4-ol) (5'')

Atoms	Coordinates (Å)			Atoms	Coordinates (Å)		
	x	y	z		x	y	z
C	2.839531	-0.77311	-0.75725	C	-2.8395	0.775054	-0.75548
C	3.851605	0.11497	-0.27504	C	-3.20732	2.008819	-1.25431
C	5.211529	-0.31979	-0.30245	H	-2.43617	2.680622	-1.61419
C	5.545664	-1.5972	-0.81118	C	-4.55587	2.423799	-1.28215
H	6.583314	-1.90458	-0.81962	H	-4.80366	3.403929	-1.67324
C	4.555844	-2.42072	-1.2876	C	-5.54567	1.599177	-0.80758
H	4.80362	-3.39999	-1.68086	H	-6.58332	1.906572	-0.81526
C	3.207309	-2.00576	-1.25886	C	-5.2115	0.320647	-0.30172
H	2.436145	-2.67671	-1.62031	C	-3.85157	-0.11417	-0.27535
C	4.423501	2.149495	0.603187	C	-4.42342	-2.15072	0.5982
H	4.104412	3.129428	0.951381	H	-4.10433	-3.13148	0.944067
C	5.796223	1.825066	0.645178	C	-5.79616	-1.82645	0.640845
H	6.515934	2.543416	1.024642	H	-6.51587	-2.54574	1.018524
C	6.192159	0.588806	0.18763	C	-6.19211	-0.5891	0.186259
C	1.410002	-0.36931	-0.73489	N	3.478814	1.349295	0.165534
C	0.689756	-0.18173	-1.88665	N	1.208626	-0.34458	1.720713
H	1.188364	-0.30259	-2.84197	N	-1.20882	0.339737	1.721549
C	-0.68961	0.186855	-1.88619	N	-3.47874	-1.34948	0.162437
H	-1.18815	0.310337	-2.84121	O	7.483513	0.170815	0.168153
C	-1.40997	0.371253	-0.73398	H	8.049192	0.864986	0.523393
C	-0.70092	0.187226	0.49751	O	-7.48347	-0.17108	0.16779
C	0.700838	-0.1887	0.497044	H	-8.04905	-0.8658	0.52211
				S	-0.00013	-0.00385	2.773265

8-[7-(4-Hydroxyquinolin-8-yl)benzo[*c*][1,2,5]thiadiazol-4-yl]quinolin-4(*1*H)-one (5''')

Atoms	Coordinates (Å)			Atoms	Coordinates (Å)		
	x	y	z		x	y	z
C	-2.84023	-0.98341	-0.29274	H	-4.48749	2.823885	1.496588
C	-3.93168	-0.13641	0.050782	C	-6.07256	1.532409	0.867429
C	-5.25876	-0.54416	-0.20955	H	-6.87177	2.185804	1.189998
C	-5.4974	-1.75916	-0.86257	C	-6.42445	0.287853	0.206241
H	-6.53083	-2.02656	-1.04639	C	-1.41969	-0.74288	0.088737
C	-4.44513	-2.56359	-1.2529	C	-0.68744	-1.75891	0.665659
H	-4.62479	-3.49431	-1.77796	H	-1.19157	-2.68877	0.900986
C	-3.13555	-2.17439	-0.95804	C	0.701835	-1.66516	0.964571
H	-2.31211	-2.81093	-1.26011	H	1.190365	-2.53051	1.397952
C	-4.77628	1.880931	1.047449	C	1.448036	-0.54586	0.69585
				C	0.733711	0.561823	0.138339
				C	-0.68764	0.469075	-0.15543

C	2.897089	-0.46625	1.005617
C	3.338779	-0.57294	2.309629
H	2.611545	-0.69777	3.104087
C	4.709929	-0.49562	2.633063
H	5.018166	-0.57357	3.669153
C	5.646228	-0.31203	1.645574
H	6.700688	-0.24389	1.879256
C	5.23471	-0.215	0.29534
C	3.851393	-0.2979	-0.04638
C	4.295845	-0.10173	-2.28234
H	3.916967	-0.06797	-3.30125
C	5.685583	0.012701	-2.06793
H	6.358935	0.137347	-2.90973
C	6.157911	-0.04726	-0.77625
N	-3.7338	1.076439	0.687142
N	-1.19223	1.607893	-0.64327
N	1.251067	1.761902	-0.12719
N	3.401938	-0.25294	-1.33152
O	-7.58121	-0.06628	-0.00682
O	7.470604	0.039374	-0.44791
H	7.996094	0.1494	-1.24798
S	0.043199	2.689677	-0.71444
H	-2.81491	1.492275	0.617163

8,8'-(Benzo[*c*][1,2,5]thiadiazole-4,7-diyl)bis(4-hydroxyquinolin-1-ium) $[5H_2^{2+}]$

Atoms	Coordinates (Å)		
	x	y	z
C	-2.91105	-0.85154	-0.57627
C	-3.98483	-0.11593	0.00708
C	-5.33526	-0.47833	-0.25561
C	-5.62634	-1.51693	-1.16551
H	-6.65837	-1.77595	-1.35771
C	-4.59484	-2.16690	-1.79772
H	-4.79970	-2.94453	-2.52252
C	-3.26482	-1.83854	-1.49451
H	-2.47568	-2.37805	-2.00249
C	-4.75405	1.61853	1.46408
H	-4.45651	2.45593	2.08173
C	-6.07743	1.26519	1.30019
H	-6.84967	1.81491	1.82269
C	-6.38324	0.22738	0.42090
C	-1.46282	-0.72975	-0.23032
C	-0.70079	-1.87526	-0.11170
H	-1.18590	-2.84067	-0.18284
C	0.70081	-1.87524	0.11164
H	1.18594	-2.84064	0.18285
C	1.46282	-0.72972	0.23022
C	0.72173	0.48980	0.08996
C	-0.72176	0.48978	-0.09007
C	2.91103	-0.85150	0.57622
C	3.26474	-1.83855	1.49442
H	2.47557	-2.37809	2.00234
C	4.59475	-2.16697	1.79766
H	4.79956	-2.94464	2.52243
C	5.62629	-1.51700	1.16552
H	6.65830	-1.77606	1.35773
C	5.33526	-0.47834	0.25566
C	3.98485	-0.11590	-0.00707
C	4.75417	1.61860	-1.46397
H	4.45667	2.45604	-2.08159
C	6.07753	1.26521	-1.30005
H	6.84981	1.81493	-1.82250
C	6.38329	0.22736	-0.42079
N	-3.76896	0.95161	0.85308
N	-1.23065	1.72629	-0.12655
N	1.23059	1.72632	0.12643
N	3.76904	0.95168	-0.85303
O	-7.61850	-0.16475	0.15297
H	-8.28502	0.34597	0.63346
O	7.61852	-0.16482	-0.15285
H	8.28508	0.34590	-0.63330
S	-0.00005	2.79625	-0.00004
H	-2.83172	1.35868	0.85730
H	2.83181	1.35878	-0.85726

8,8'-(Benzo[*c*][1,2,5]thiadiazole-4,7-diyl)bis(4-hydroxyquinolin-1-ium) with two trifluoroacetates and two TFA molecules ($[5H_2^{2+}\ 2TFA^-]\cdot 2TFA$)

Atoms	Coordinates (Å)		
	x	y	z
**C**	1.57201	-2.16677	-1.43442
**C**	2.88861	-2.42743	-0.97290
**C**	3.40872	-3.75085	-1.00746
**C**	2.61870	-4.80158	-1.52613
**H**	3.02572	-5.80328	-1.54086
**C**	1.35497	-4.53539	-1.99185
**H**	0.73586	-5.33384	-2.38182
**C**	0.83686	-3.22781	-1.93605

**H**	-0.18138	-3.04374	-2.25394
**C**	4.92352	-1.64154	-0.04212
**H**	5.47904	-0.77355	0.29183
**C**	5.47382	-2.91579	-0.01658
**H**	6.47484	-3.04692	0.37451
**C**	4.72659	-3.97345	-0.50646
**C**	0.93344	-0.82927	-1.34497
**C**	0.50432	-0.14639	-2.45376
**H**	0.71870	-0.54611	-3.43822
**C**	-0.24013	1.06564	-2.36570
**H**	-0.55611	1.54262	-3.28609
**C**	-0.60162	1.61975	-1.16517
**C**	-0.14894	0.95705	0.01798
**C**	0.62415	-0.25974	-0.07135
**C**	-1.42651	2.85226	-1.06839
**C**	-0.91605	4.07874	-1.45367
**H**	0.09852	4.12130	-1.82732
**C**	-1.66564	5.26532	-1.33297
**H**	-1.22226	6.20544	-1.63774
**C**	-2.93633	5.23761	-0.81287
**H**	-3.51759	6.14228	-0.69781
**C**	-3.50556	4.00511	-0.41991
**C**	-2.75239	2.80842	-0.56105
**C**	-4.58294	1.54262	0.25999
**H**	-4.95196	0.54540	0.47245
**C**	-5.35790	2.67916	0.45157
**H**	-6.36164	2.55949	0.84059
**C**	-4.82948	3.91142	0.10869
**N**	3.69590	-1.41913	-0.49976
**H**	3.40264	-0.41019	-0.49290
**N**	0.96444	-0.76299	1.11383
**N**	-0.38112	1.34792	1.27053
**N**	-3.34522	1.61443	-0.22150
**H**	-2.86589	0.67909	-0.36899
**O**	5.17823	-5.23488	-0.54228
**H**	6.07087	-5.28360	-0.17876
**O**	-5.50737	5.06194	0.23708
**H**	-6.38553	4.89285	0.59982
**S**	0.34378	0.24824	2.24392
**C**	-3.36920	-1.73975	-0.28104
**C**	-2.99019	-3.22499	-0.54712
**O**	-4.50343	-1.51198	0.18431
**O**	-2.47292	-0.92073	-0.56411
**F**	-1.93226	-3.59418	0.20552
**F**	-2.63391	-3.40559	-1.84515
**F**	-3.99200	-4.07638	-0.28816
**C**	-7.58066	-1.16028	1.02957
**C**	-9.05539	-1.50668	1.35767
**O**	-6.90756	-2.24819	0.76046
**H**	-5.94696	-2.04615	0.52867
**O**	-7.19474	-0.01582	1.03790
**F**	-9.13343	-2.37654	2.37908
**F**	-9.73697	-0.40575	1.69470
**F**	-9.65958	-2.06115	0.29037
**C**	3.92379	2.31958	-0.41223
**C**	3.00573	3.53405	-0.74092
**O**	3.32484	1.22774	-0.37793
**O**	5.12401	2.60027	-0.22676
**H**	6.09577	1.63569	0.20432
**F**	2.40016	3.35854	-1.94822
**F**	3.65184	4.70220	-0.78970
**F**	2.01683	3.65110	0.17478
**C**	7.51159	1.08179	1.50838
**C**	8.38873	-0.16725	1.79594
**O**	6.76008	0.85520	0.45602
**O**	7.58206	2.06127	2.19669
**F**	9.07389	-0.04061	2.92980
**F**	9.26061	-0.39585	0.79612
**F**	7.61410	-1.28581	1.90850

8,8'-(Benzo[*c*][1,2,5]thiadiazole-4,7-diyl)bis(4-hydroxyquinolin-1-ium) with four trifluoroacetates and four TFA molecules ([$5H_2^{2+}$ $4TFA^-$]·4TFA)

**Atoms**	**Coordinates (Å)**		
	**x**	**y**	**z**
**C**	-2.02534	1.09415	-1.59379
**C**	-3.40200	0.89783	-1.30604
**C**	-4.32241	1.96416	-1.47698
**C**	-3.85910	3.22144	-1.91917
**H**	-4.58174	4.01752	-2.03703
**C**	-2.52476	3.40711	-2.18901
**H**	-2.15806	4.37190	-2.51860
**C**	-1.61763	2.34495	-2.02687
**H**	-0.56489	2.50357	-2.22225
**C**	-5.18712	-0.52129	-0.63870
**H**	-5.45422	-1.51519	-0.30259
**C**	-6.13050	0.47025	-0.79645
**H**	-7.16803	0.26849	-0.57858
**C**	-5.72310	1.74293	-1.21627
**C**	-0.99440	0.02886	-1.45160
**C**	-0.36732	-0.51432	-2.54327
**H**	-0.69615	-0.22647	-3.53538
**C**	0.72193	-1.42729	-2.43059

H	1.16810	-1.81067	-3.34123
C	1.24190	-1.81055	-1.22190
C	0.60869	-1.28412	-0.05289
C	-0.51504	-0.37775	-0.16764
C	2.38889	-2.75540	-1.11891
C	2.18882	-4.10410	-1.35339
H	1.19032	-4.44201	-1.59619
C	3.23825	-5.03710	-1.25846
H	3.03458	-6.08604	-1.43952
C	4.50474	-4.62223	-0.92607
H	5.32828	-5.31810	-0.83977
C	4.76080	-3.25357	-0.69342
C	3.70074	-2.31575	-0.79159
C	5.21925	-0.55462	-0.31365
H	5.30702	0.52190	-0.20190
C	6.29323	-1.41725	-0.19393
H	7.28262	-1.03753	0.02095
C	6.08563	-2.78848	-0.37693
N	-3.88550	-0.31945	-0.88601
H	-3.27432	-1.14086	-0.74676
N	-0.99557	0.02506	1.00792
N	0.95368	-1.54584	1.20767
N	3.98273	-0.98468	-0.59349
H	3.24983	-0.23263	-0.67814
O	-6.53358	2.74772	-1.39325
H	-7.52853	2.50207	-1.28483
O	7.03023	-3.69413	-0.28806
H	7.94048	-3.29211	-0.07289
S	-0.08190	-0.70186	2.15620
C	-9.67055	2.34703	-2.25455
C	-11.1881	2.03432	-2.08671
O	-9.00085	2.15457	-1.17063
O	-9.27378	2.72838	-3.33602
F	-11.8835	2.24899	-3.21555
F	-11.3900	0.74557	-1.72844
F	-11.7436	2.80604	-1.12128
C	-8.89773	1.38674	2.03368
C	-9.42743	0.88325	3.40043
O	-9.85713	1.35415	1.14157
H	-9.53531	1.68875	0.23789
O	-7.75912	1.75253	1.90115
F	-10.3519	1.74152	3.89293
F	-8.44060	0.79328	4.29853
F	-10.0115	-0.32374	3.29921
C	10.42796	-2.59540	0.08170
C	10.98159	-4.05020	0.01444
O	11.28164	-1.69616	0.00873
O	9.19216	-2.49116	0.23695
F	11.17158	-4.54244	1.26173
F	12.15790	-4.13535	-0.63125
F	10.12235	-4.88621	-0.61257
C	9.98845	1.30434	0.92172
C	8.99581	2.46733	0.65397
O	10.51629	1.16566	1.99394
O	10.10602	0.57549	-0.15970
H	10.63052	-0.29396	-0.00771
F	8.88476	3.26821	1.72049
F	7.76484	1.98283	0.37410
F	9.38959	3.21703	-0.39194
C	3.01256	2.27264	-0.65916
C	2.12801	3.54743	-0.80361
O	4.23413	2.44360	-0.46603
O	2.38116	1.20543	-0.77345
F	1.09425	3.53600	0.05798
F	1.59859	3.61665	-2.05693
F	2.80724	4.69106	-0.60781
C	5.83050	4.98782	1.05959
C	6.83134	6.17133	1.11066
O	5.78882	4.49096	-0.15245
H	5.11672	3.73765	-0.22904
O	5.20468	4.66650	2.03401
F	6.29153	7.26231	0.51423
F	7.11778	6.50705	2.37587
F	7.98757	5.90178	0.48166
C	-2.96912	-4.01474	-0.34008
C	-1.69056	-4.88648	-0.52758
O	-2.77167	-2.79585	-0.43936
O	-4.01527	-4.66610	-0.11986
H	-5.23855	-4.01063	0.20582
F	-1.17199	-4.71002	-1.77543
F	-1.89884	-6.20381	-0.37775
F	-0.72538	-4.53009	0.34828
C	-6.73504	-3.76604	1.50670
C	-8.01025	-2.89378	1.67202
O	-6.14101	-3.48030	0.37693
O	-6.43011	-4.57407	2.34439
F	-8.55563	-3.05060	2.88146
F	-8.93924	-3.22381	0.75090
F	-7.73068	-1.57892	1.51165

S2.4 Additional photophysical data

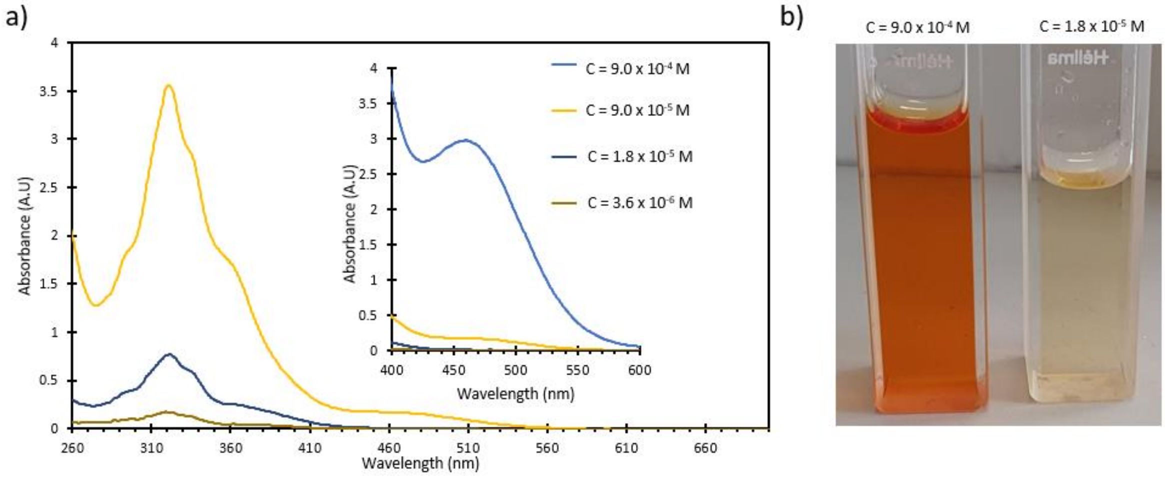

Figure S11. *a) UV-Vis absorption spectra of* ***5****H_2^{2+} dissolved in DMSO in various concentrations. Insert shows that a new peak appears at 460 nm consistent with excimer formation. b) Photograph of a concentrated (left) and dilute (right) solution of* ***5****H_2^{2+} reveals a stark colour difference between the two. The deep orange colour on the left is caused by the appearance of the new absorption peak at 460 nm.*

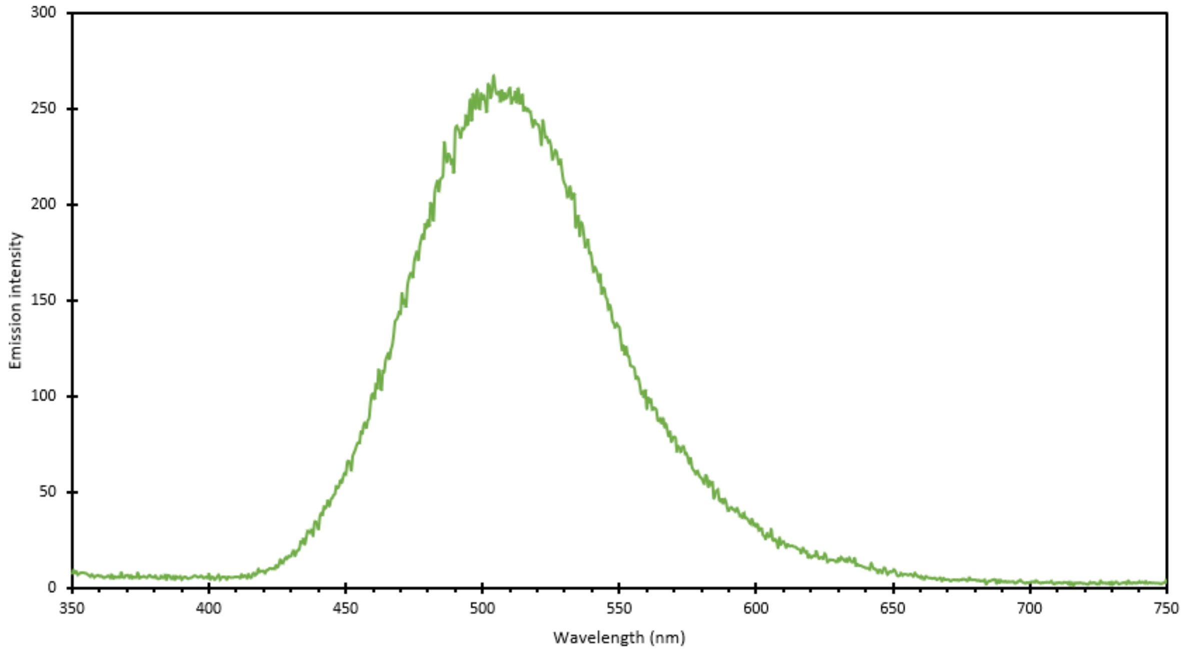

Figure S12. *Solid state fluorescence of* ***5****. Maximum emission was recorded at λ_{em} = 512 nm.*

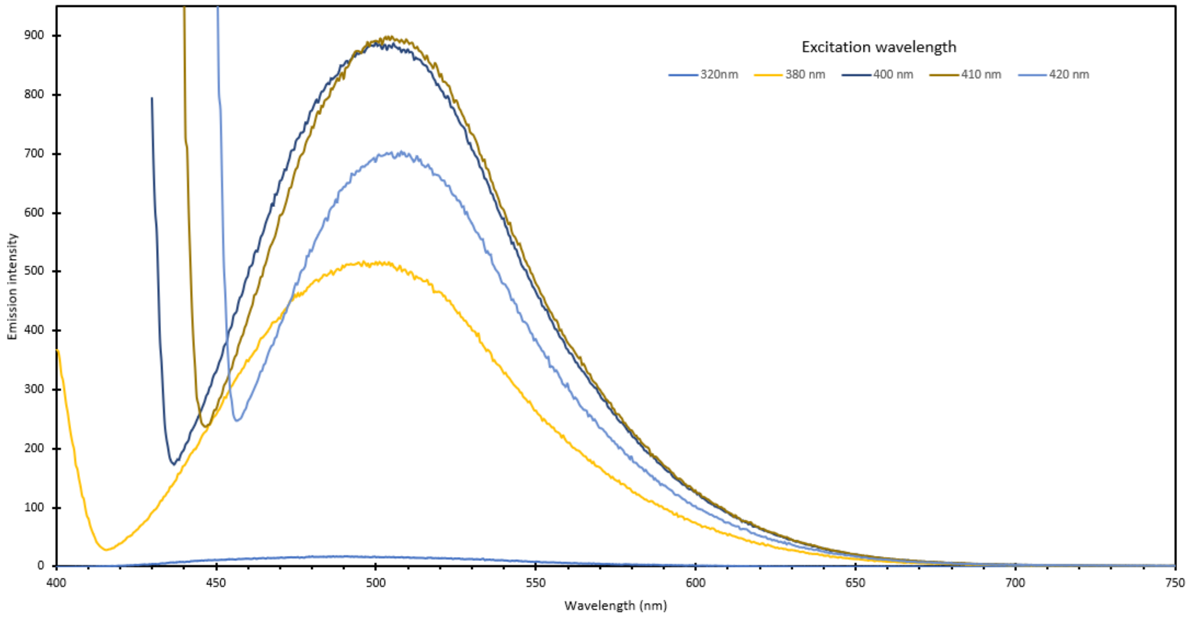

Figure S13. *Fluorescence spectra of **5**H_2^{2+} in DMSO with varying excitation wavelengths. Maximum emission intensity is observed when λ_{ex} = 410 nm.*

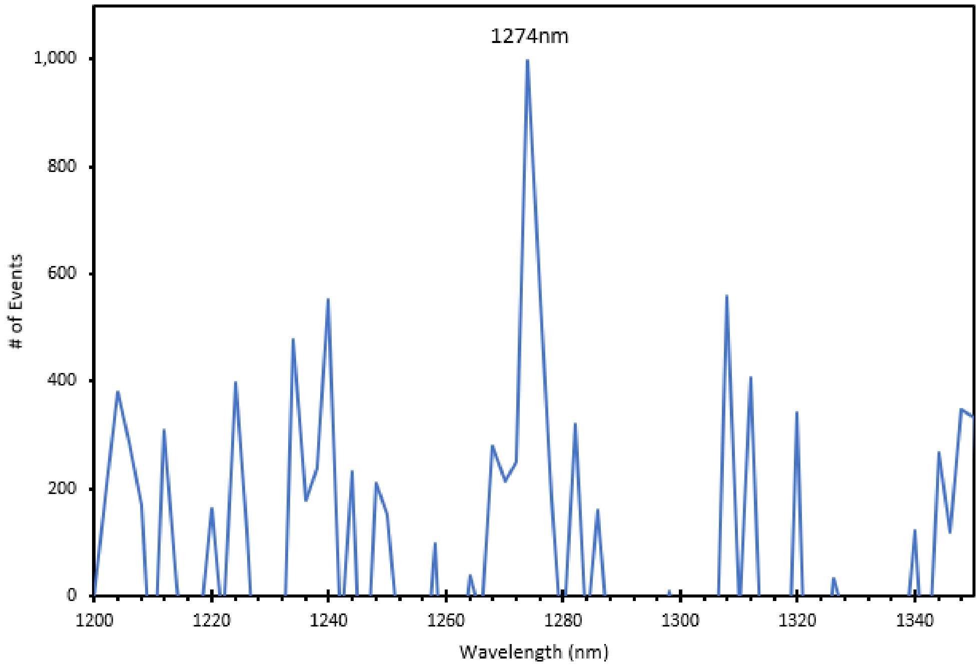

Figure S14. *IR emission spectrum of **5**H_2^{2+} in TFA/D_2O (1:1) solution shows a weak peak at 1274 nm which is attributed to $^1O_2$ phosphorescence.*

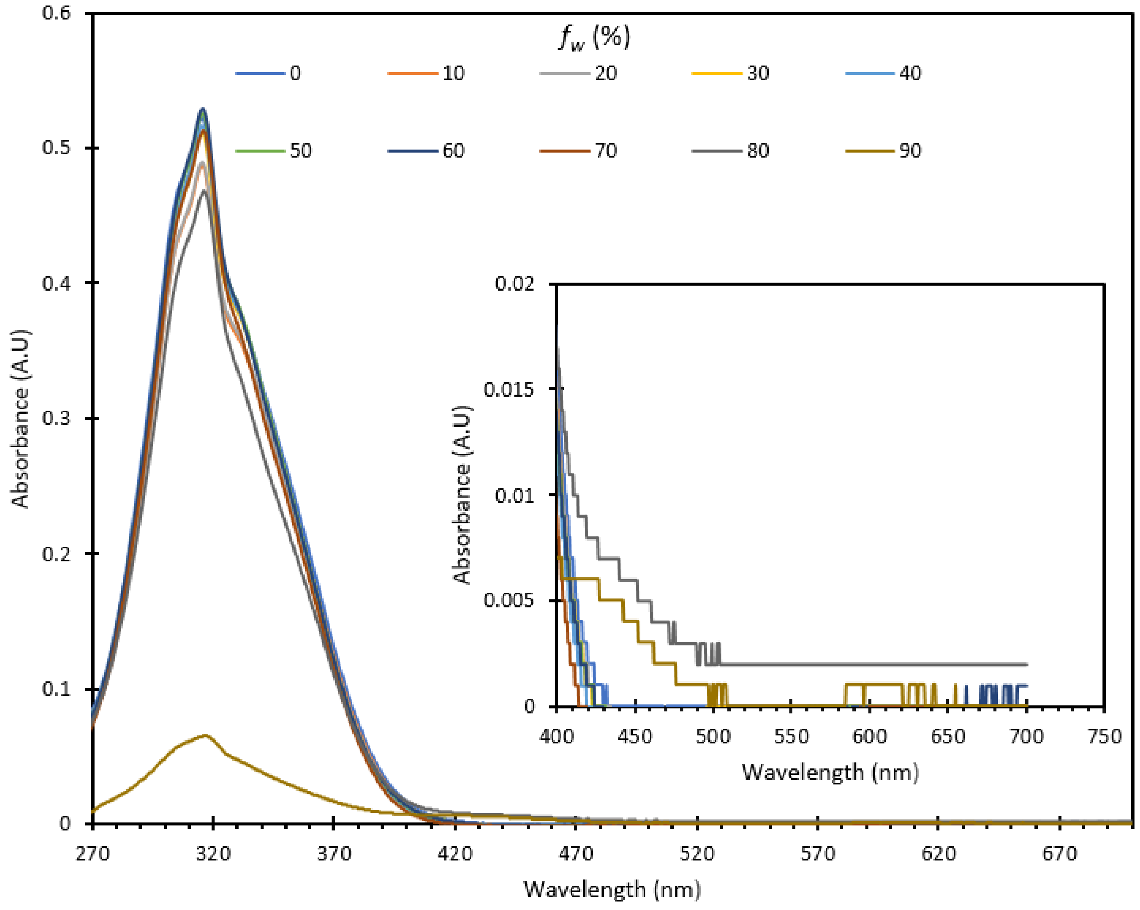

Figure S15. *UV-Vis spectra corresponding to the TFA solution series. Insert highlights the tailing-off effect caused by the production of nanoaggregates. It can be seen that at 80% water, tailing occures. At 90% the effect is not as pronounced, possibly due to sedimentation of the nanoaggregates.*

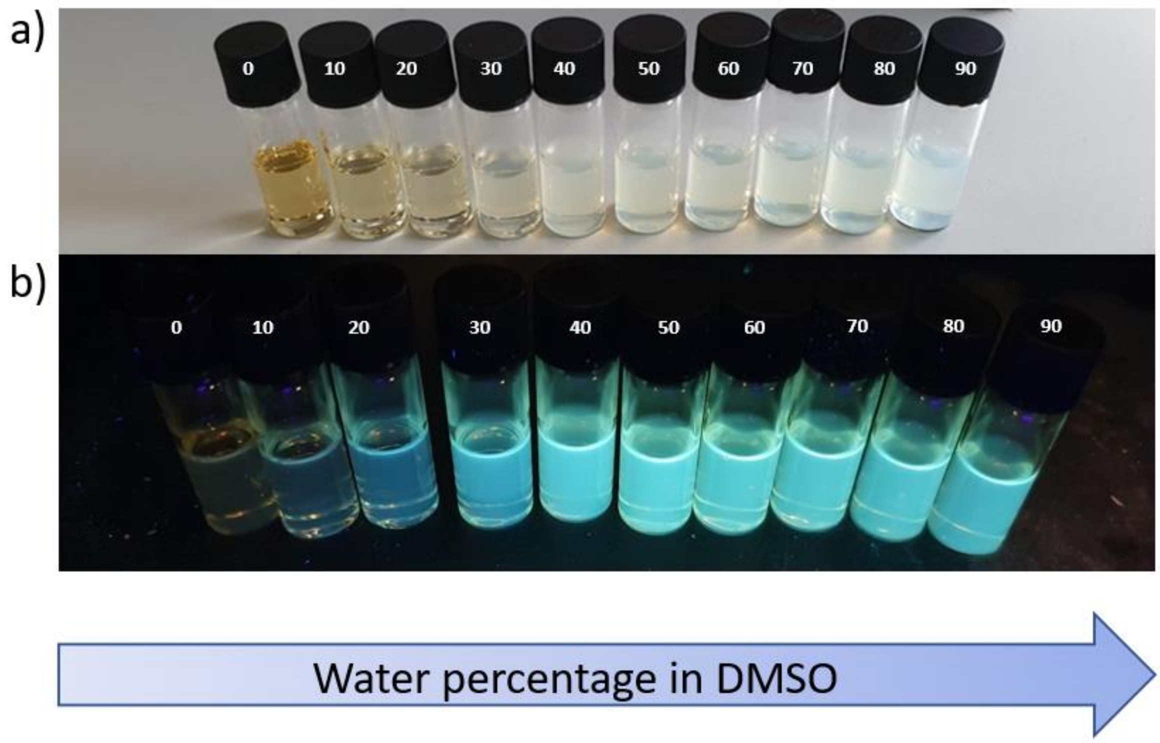

Figure S16. *Picture of series in increasing water percentage compared to DMSO. Concentration was kept constant at 2.05 × 10^{-4} M. a) Illumination with visible light shows that visible cloudiness occurs when water reaches 20%. b) Illumination with far UV light shows the AIE effect in action; When* ***5***H_2^{2+} *is fully dissolved in DMSO (0% vial), no emission is visible, but as the water percentage increases, a green-blue emission appears, originating from the solid that is precipitating out of solution.*

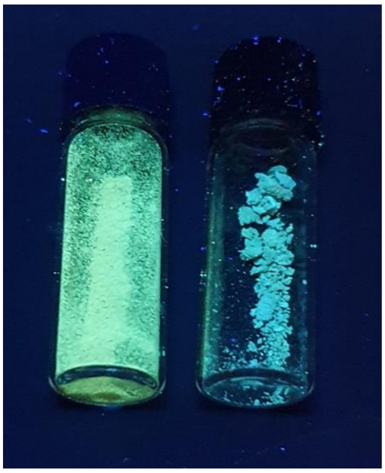

Figure S17. *Left: Solid* ***5*** *under far UV illumination.* λ_{em} *= 512 nm. Right: Solid* ***5***H_2^{2+} *under far UV illumination.* λ_{em} *= 485 nm.*

S2.5 Continuous flow set-up and cycle data

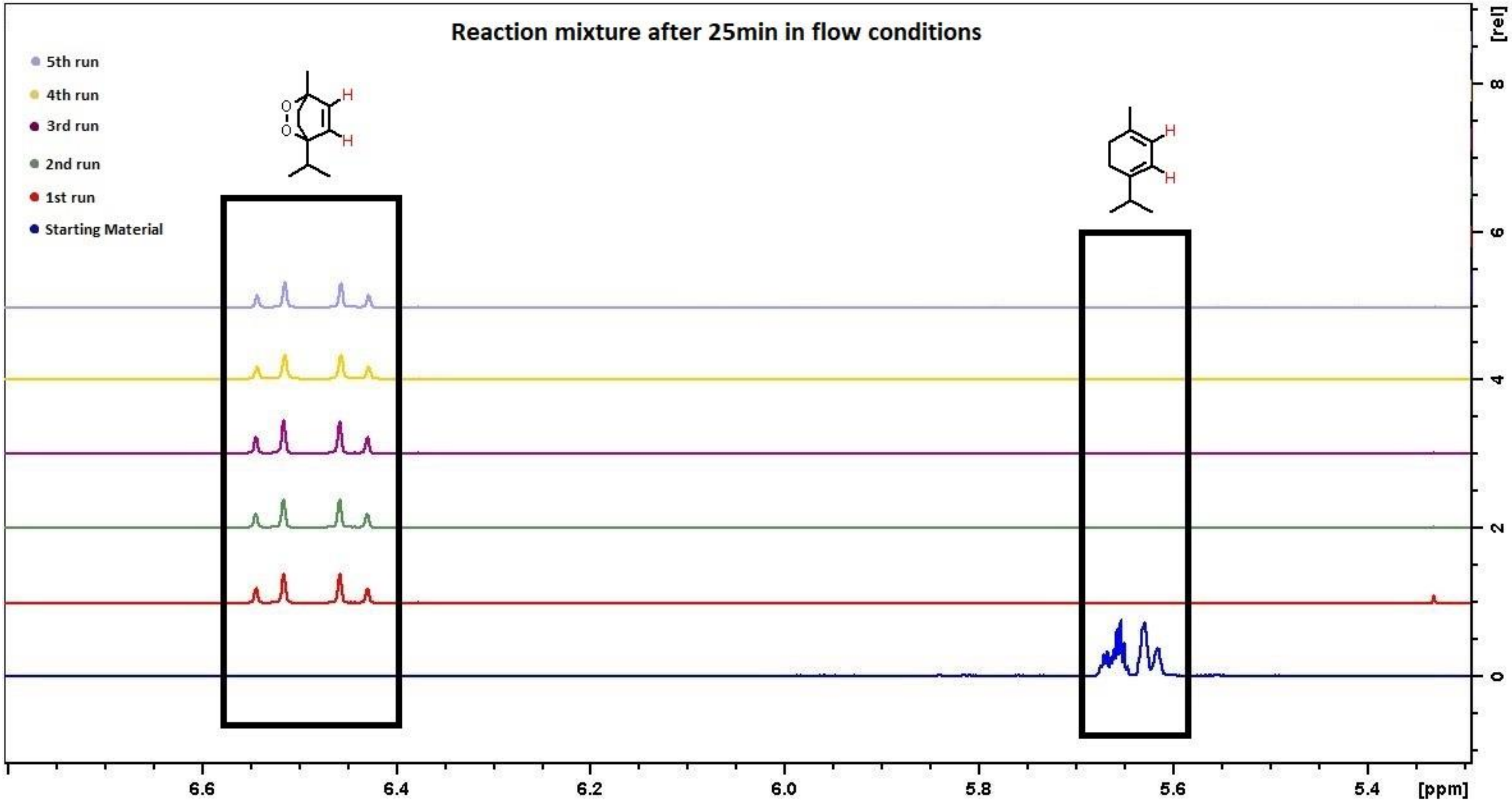

Figure S18. *Stacked 300 MHz* ^{1}*H NMR spectra illustrating the conversion to ascaridole after each run. The conversion was monitored by the disappearance of the alkene protons of the α-terpinene starting material (5.6 - 5.7 ppm range) and the subsequent appearance of the alkene protons of the product (6.4 – 6.6 ppm range).*

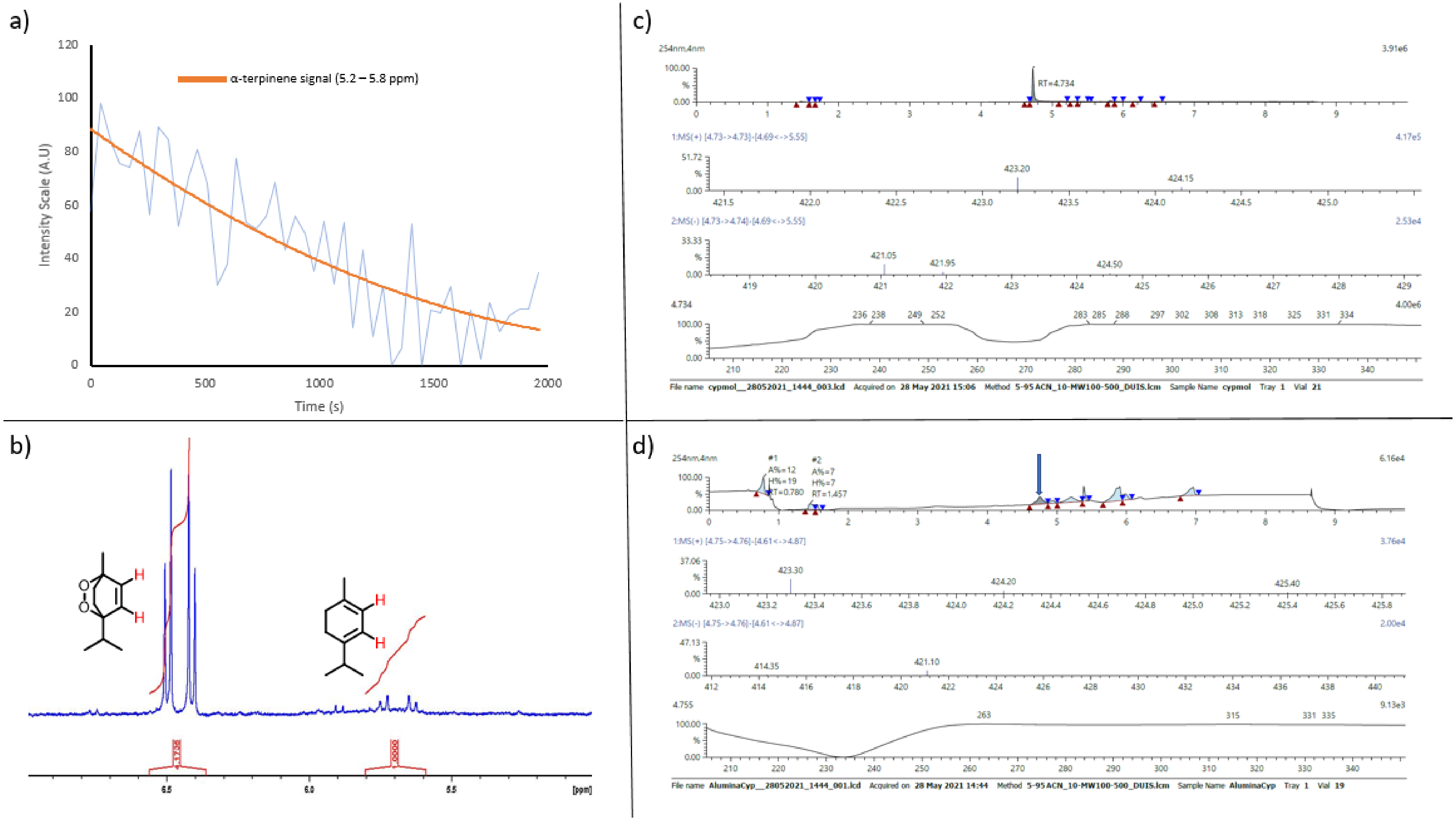

Figure S19. *a) Photosensitisation of* ***5*** *(1 mol%) for $^1O_2$ production under flow conditions. The graph shows benchtop ^{1}H NMR monitoring of a-terpinene disappearance over time, when an alumina column support is used instead of silica. b) 400 MHz ^{1}H NMR spectrum of the reaction mixture after 2000 s shows 80.7% conversion to ascaridole. c) LC-MS trace of pristine* ***5*** *(sample was dissolved in DMSO and filtered prior to injection). Retention time (RT) was 4.734 min. d) LC-MS trace of alumina supported* ***5*** *reaction mixture after 2000 s of irradiation shows the prescence of leached* ***5****. The sample was obtained by removing the reaction solvent under vacuum and dissolving the resulting residue in DMSO.*

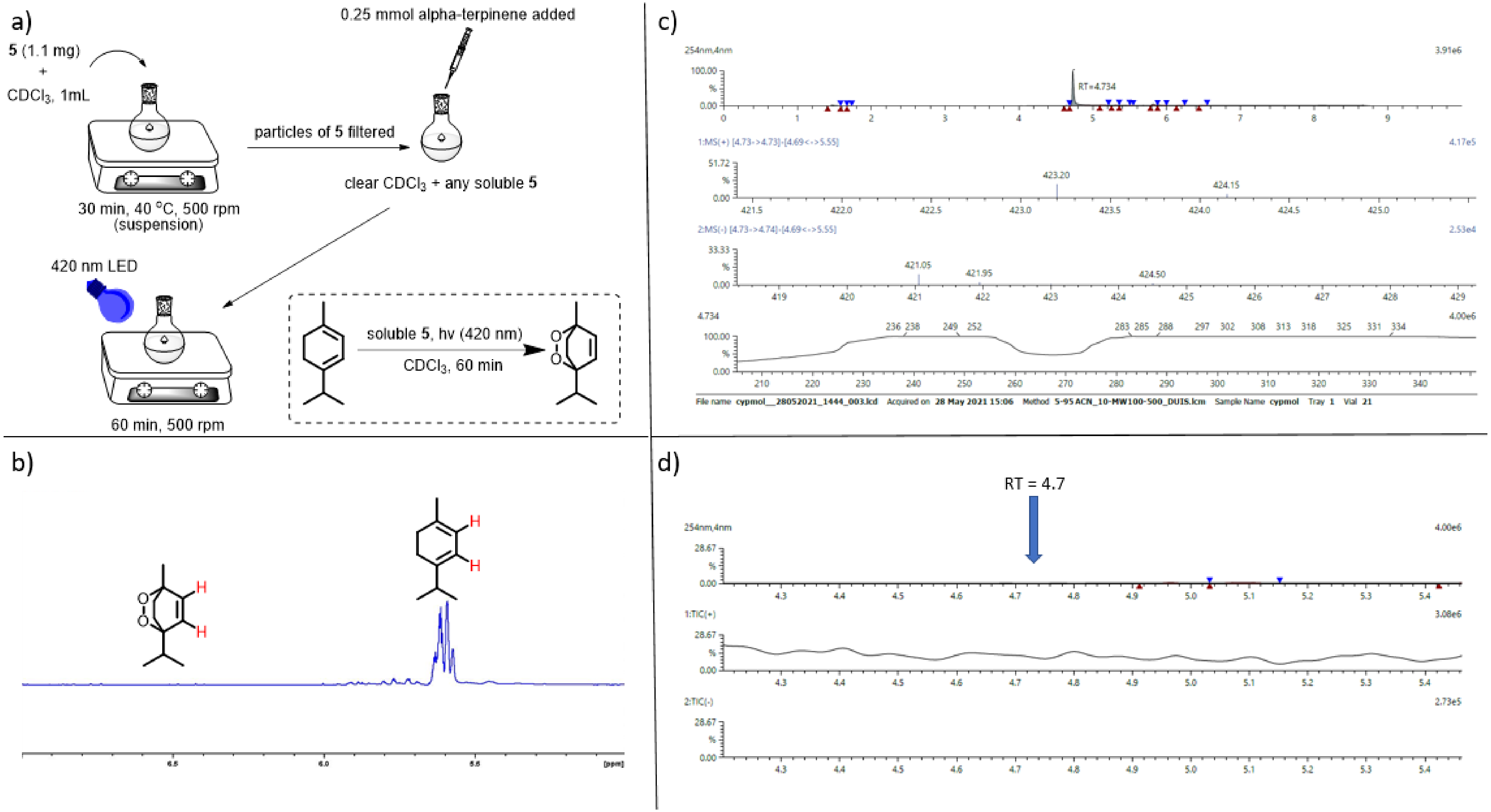

__Figure S20.__ a) Schematic of procedure that was conducted to test whether trace quantities of soluble __5__ can photocatalyse the production of ascaridole from α-terpinene. b) 300 MHz ^{1}H NMR spectrum of the reaction mixture after 60 min shows no conversion to ascaridole, confirming that the original flow reaction is indeed heterogeneously mediated. c) LC-MS trace of pristine __5__ (sample was dissolved in DMSO and filtered prior to injection). Retention time (RT) was 4.734 min. d) LC-MS trace of filtered $CDCl_3$ solution that was stirred with solid __5__ (a). No compound __5__ was detected.

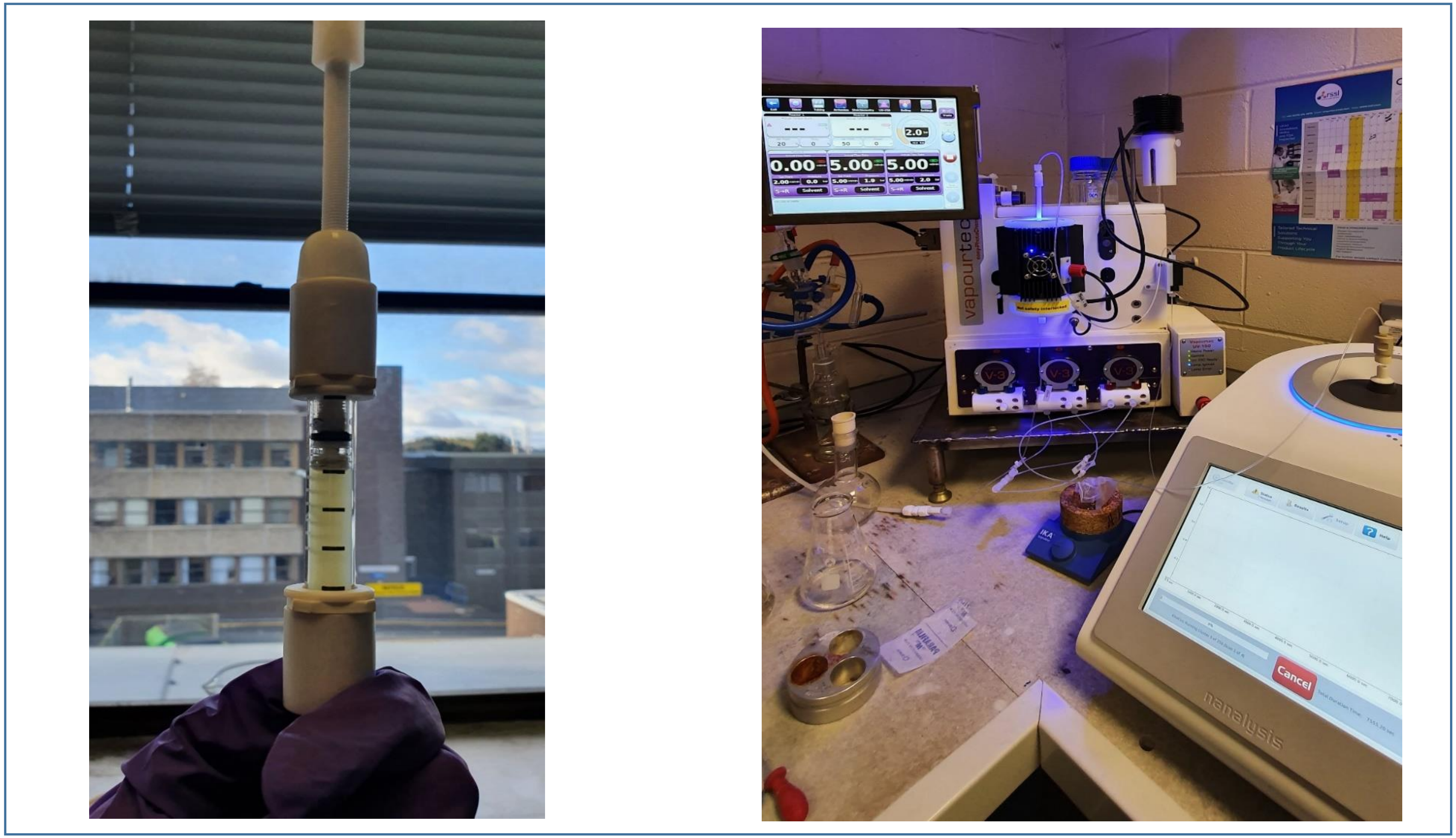

Figure S21*. a) A loaded borosilicate glass column fixed bed reactor with silica and* ***5****. The column is primed with* $CDCl_3$*. b) Picture depicting the flow reactor set-up that was used during the photosensitisation experiments.*

S2.6 X-Ray powder diffraction (XRD) of **5**

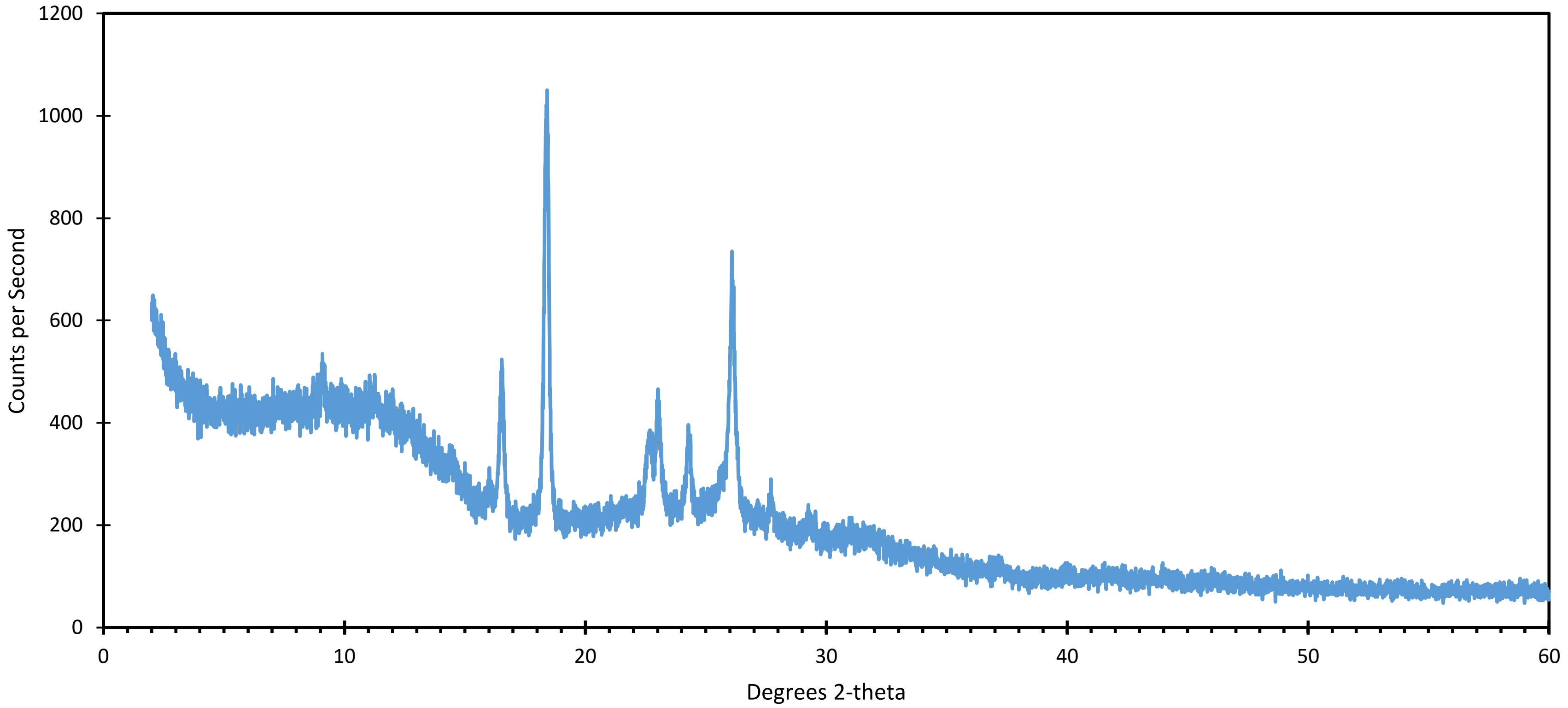

Figure S22. *XRD diffractogram of compound* ***5*** *directly after its synthesis shows the prescence of a semi-crystalline solid.*

S2.7 Solubility of **5** in various organic solvents

Table S3. Solubility of **5** in various organic solvents

Number	Solvent	Solvent Relative Polarity[4]	Solubility[a]
**1**	Water	1.000	Negligible
**2**	Trifluoroacetic acid	N/A	Soluble
**3**	Methanol	0.762	Negligible
**4**	Acetonitrile	0.460	Negligible
**5**	Dimethylsulfoxide	0.444	Partially soluble
**6**	Acetone	0.355	Negligible
**7**	Methylene Chloride	0.309	Negligible
**8**	Chloroform	0.259	Negligible
**9**	Ethyl Acetate	0.228	Negligible
**10**	Tetrahydrofuran	0.207	Negligible
**11**	Diethyl Ether	0.117	Negligible
**12**	Toluene	0.099	Negligible
**13**	n-Hexane	0.009	Negligible

[a] As determined by HPLC analysis of a filtered solution resulting from stirring 1.1 mg of **5** in 1 mL of solvent.

S2.8 $^1O_2$ generation efficiencies of **5** and $5H_2^{2+}$ under indentical conditions

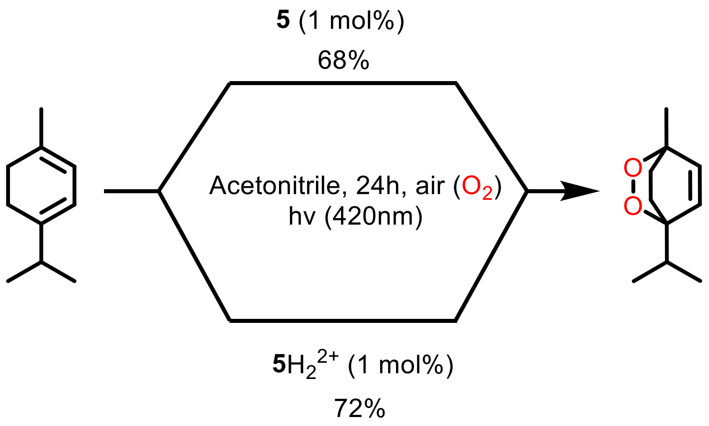

Figure S22. *α-terpinene (0.25 mmol) conversion to ascaridole under batch conditions. Photocatalysts* ***5*** *and* $5H_2^{2+}$ *exhibited similar performance under identical conditions.*

S2.9 Aerobic oxidative hydroxylation of phenylboronic acid (HPLC data)

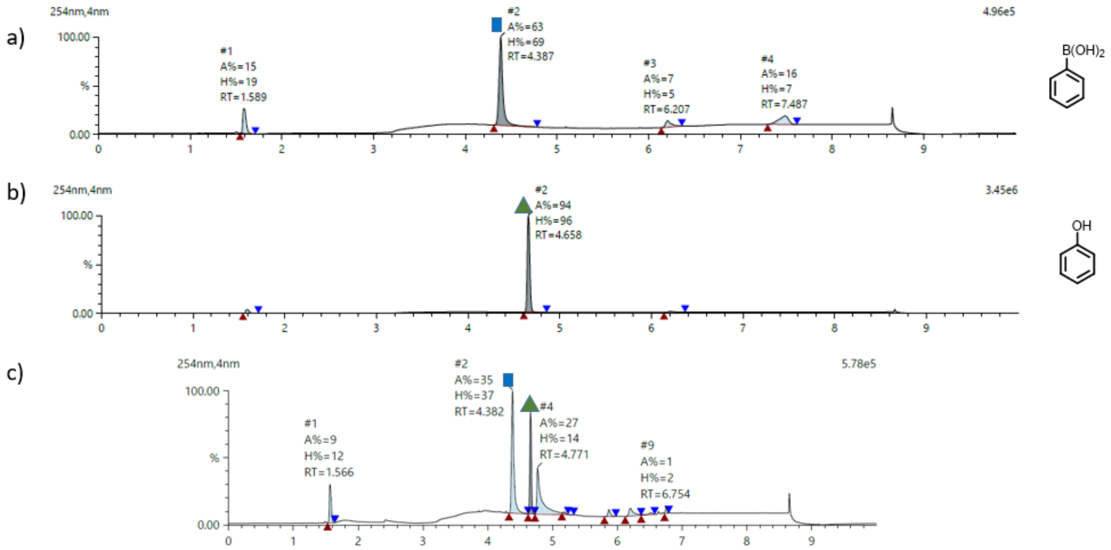

Pk#	RT	Area	Area %	Height	Height %
1	1.566	344,783	8.738	165,785	11.562
2	4.382	1,367,421	34.657	526,630	36.728
3	4.661	790,589	20.037	437,424	30.507
4	4.771	1,082,915	27.446	195,851	13.659
5	5.184	15,399	0.390	6,482	0.452
6	5.871	81,150	2.057	30,604	2.134
7	6.203	171,678	4.351	33,841	2.360
8	6.515	42,771	1.084	8,370	0.584
9	6.754	48,929	1.240	28,883	2.014
**Totals:**		**3,945,633**	**100.000**	**1,433,870**	**100.000**

Figure S24. *HPLC chromatograms of a) Neat phenylboronic acid. b) Neat phenol. c)Reaction mixture when* ***5*** *was used as a photoredox catalyst for the hydroxylation of phenylboronic acid to phenol.*

S3 NMR spectra of compounds **1**, **2**, **5** and **5**H_2^{2+}

5-(((2-iodophenyl)amino)methylene)-2,2-dimethyl-1,3-dioxane-4,6-dione

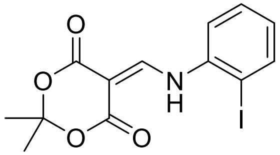

1

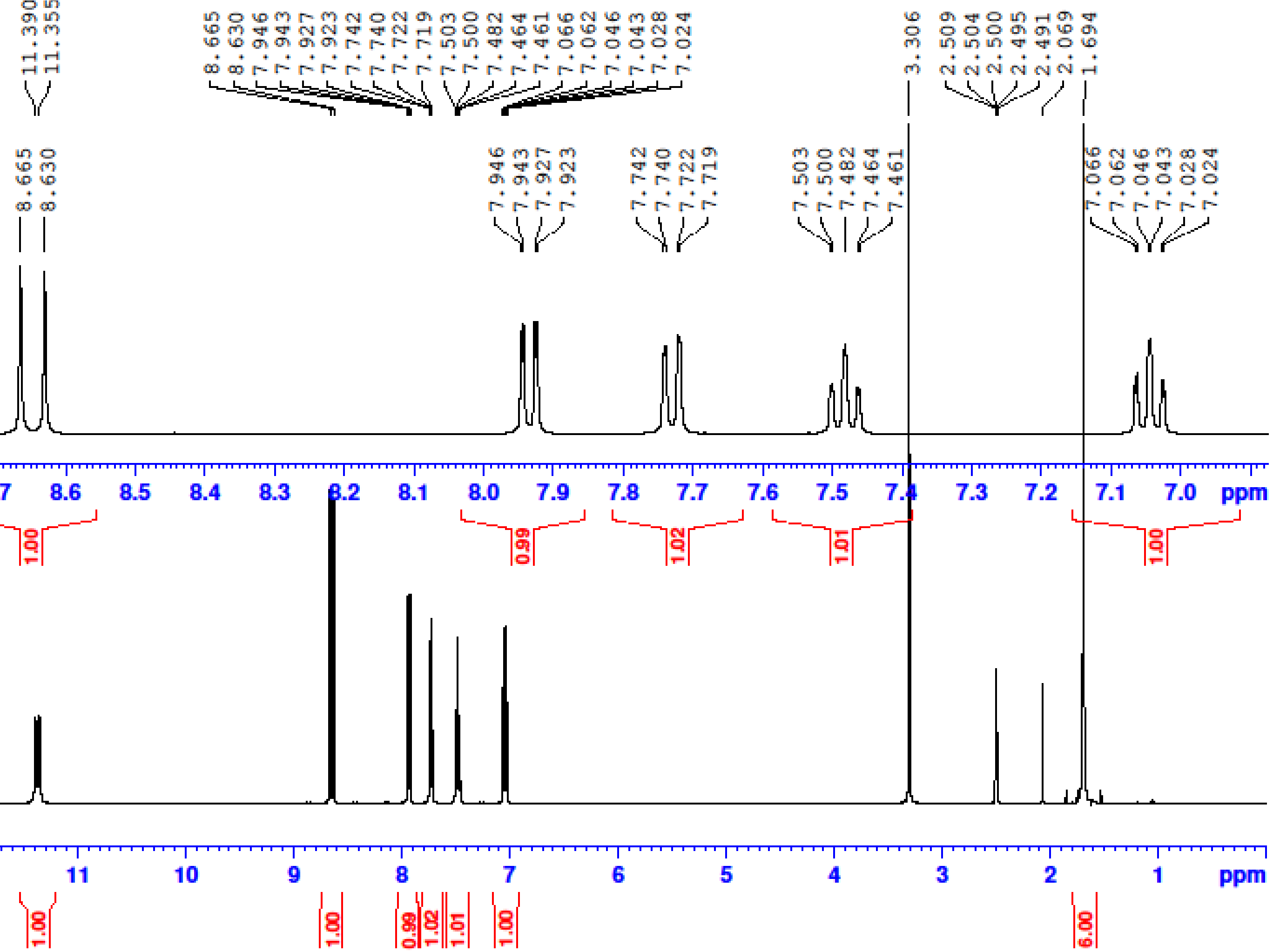

5-(((2-iodophenyl)amino)methylene)-2,2-dimethyl-1,3-dioxane-4,6-dione

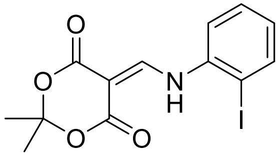

1

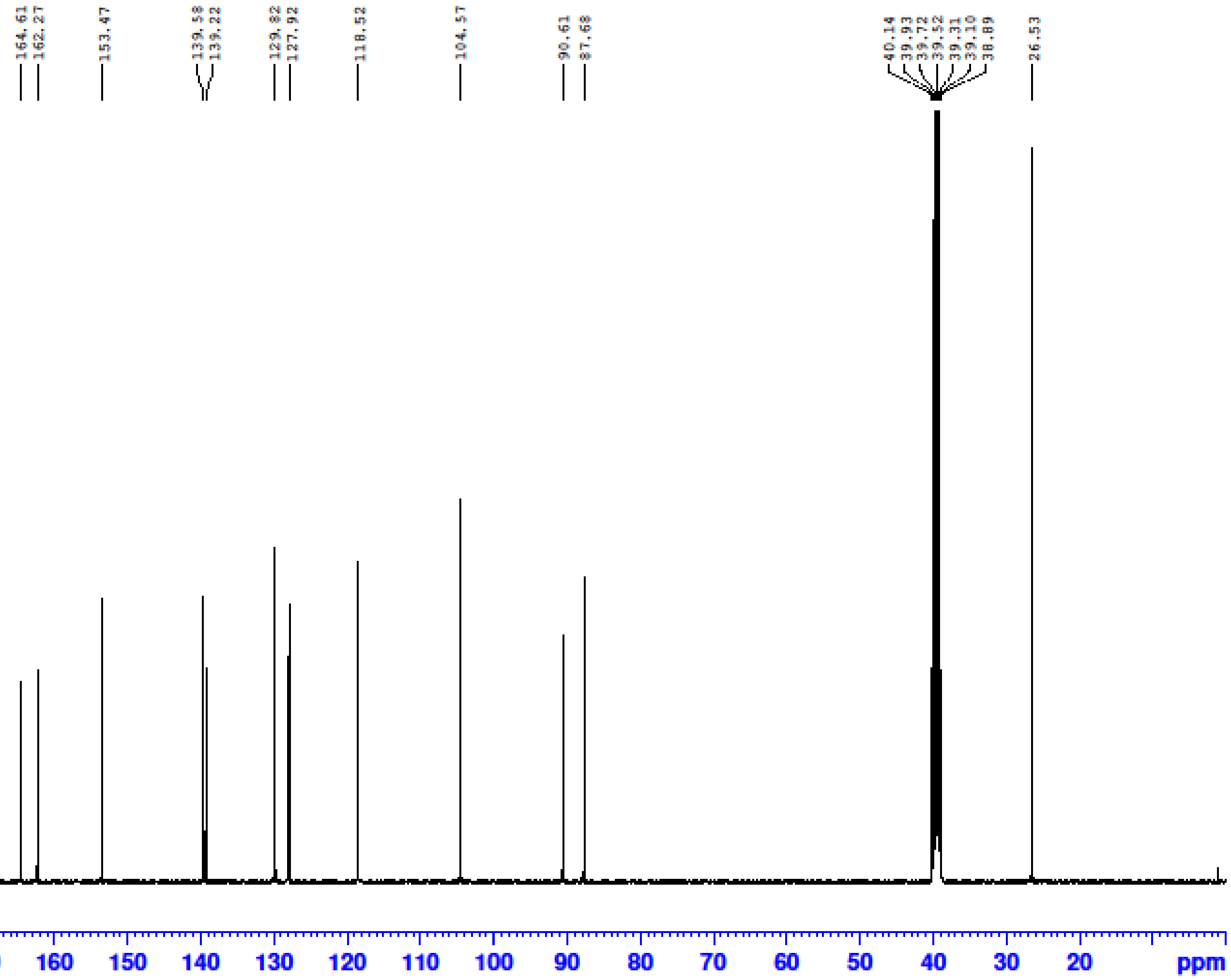

8-iodoquinolin-4(1H)-one

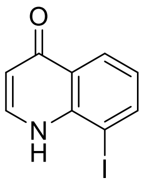

2

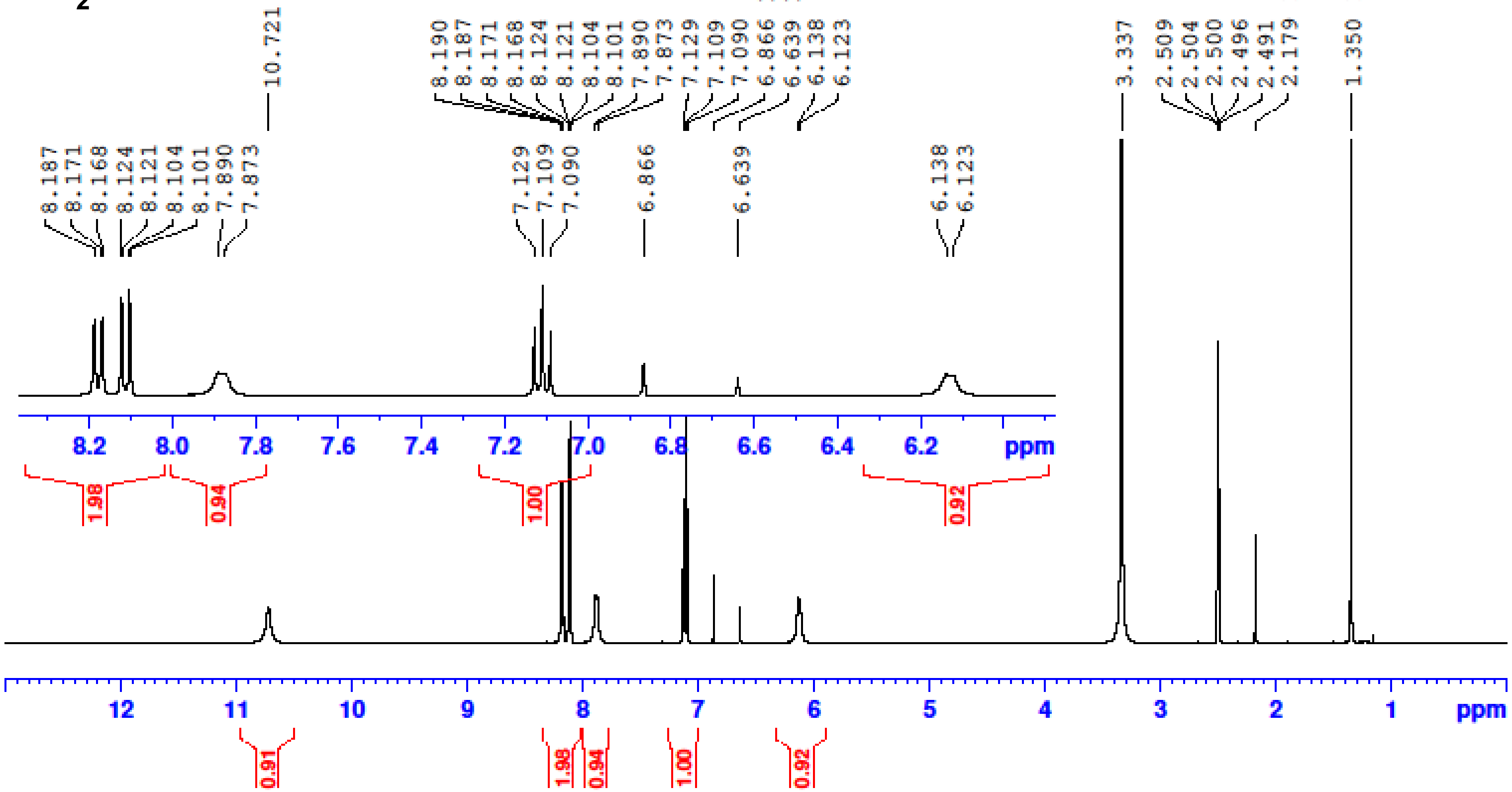

8-iodoquinolin-4(1H)-one

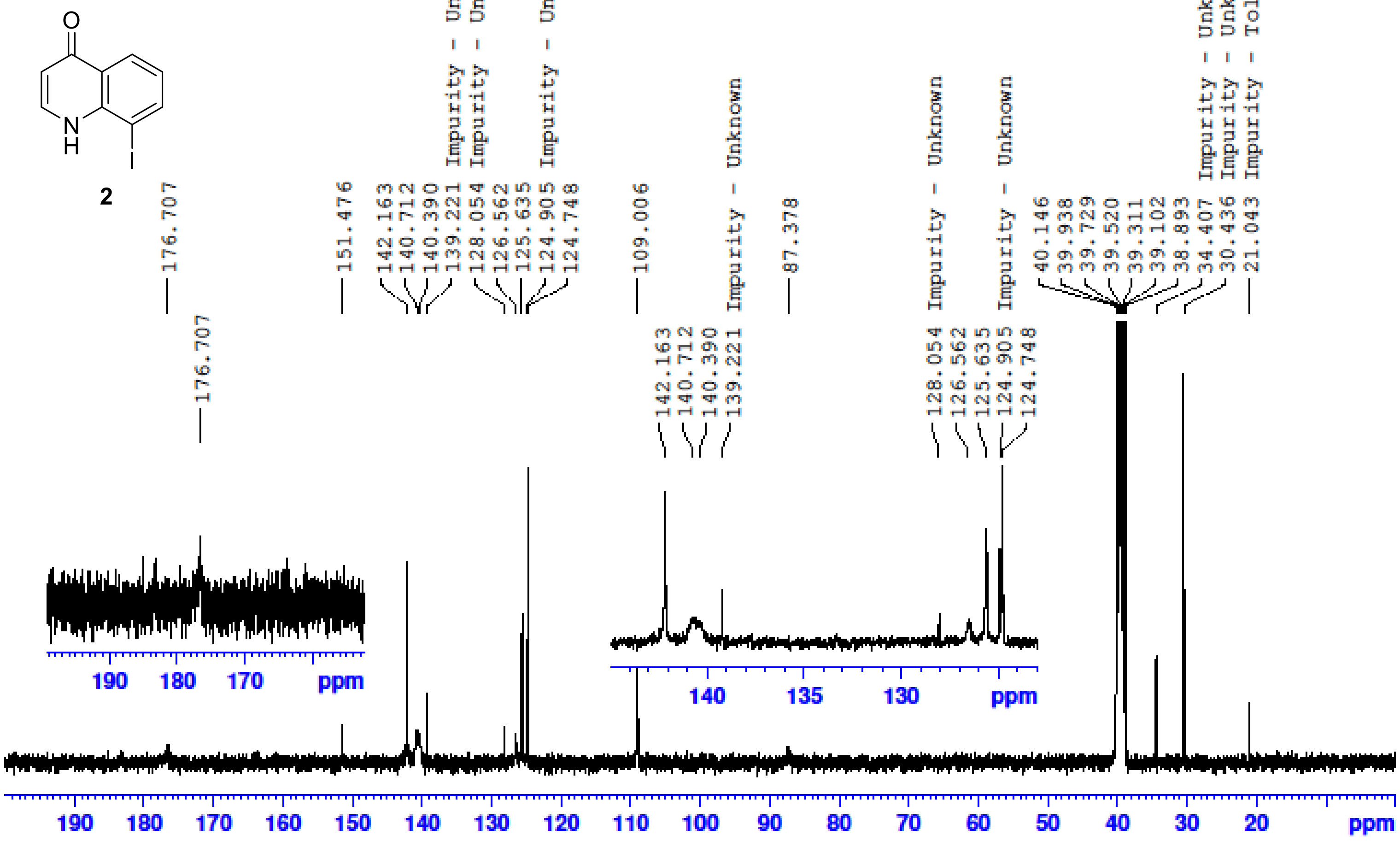

8,8'-(benzo[c][1,2,5]thiadiazole-4,7-diyl)bis(quinolin-4-ol)

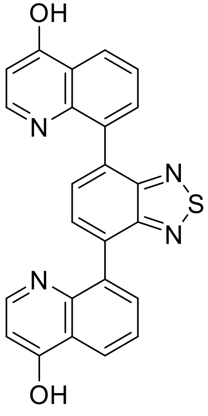

5

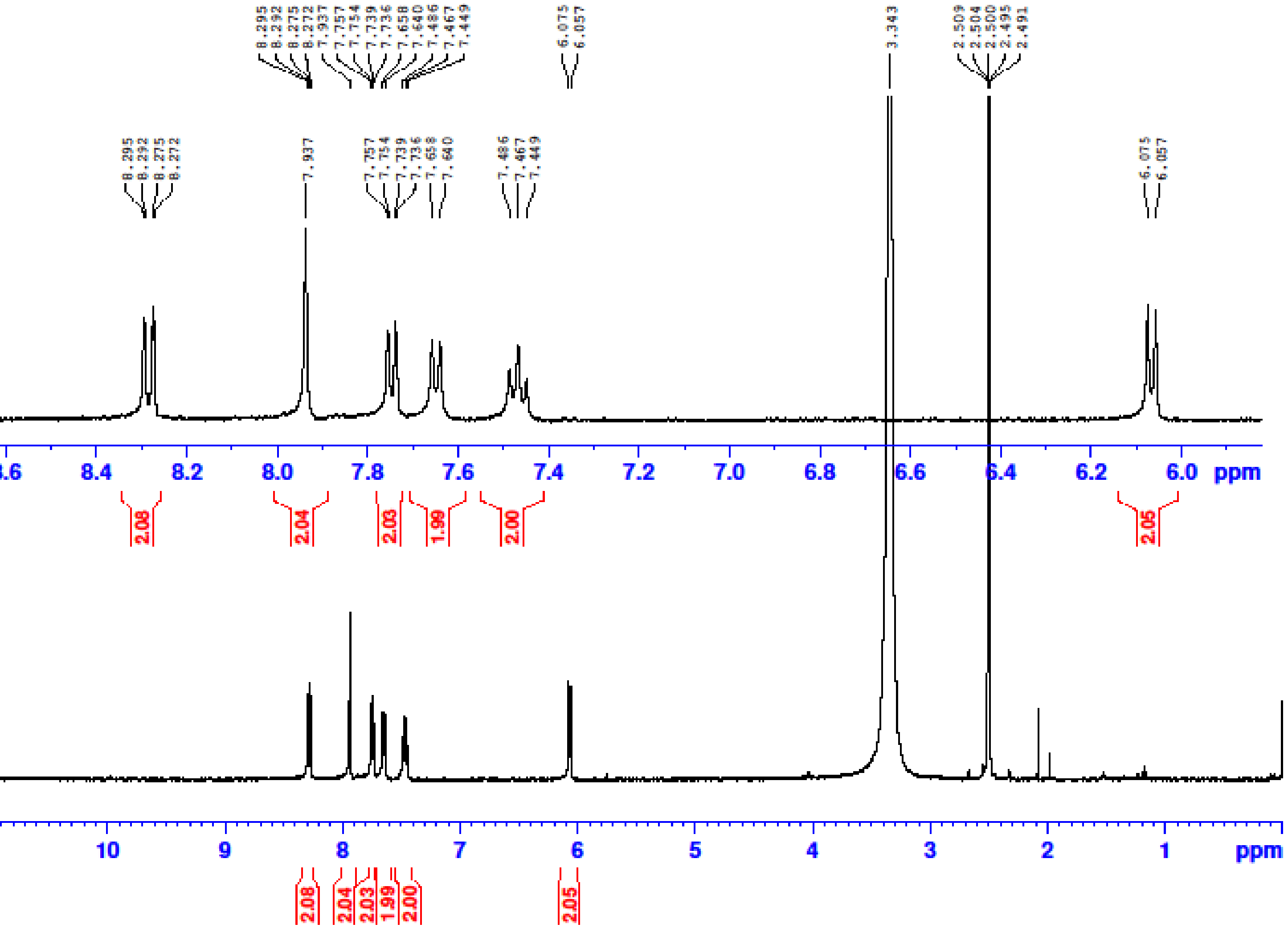

8,8'-(benzo[c][1,2,5]thiadiazole-4,7-diyl)bis(quinolin-4-ol)

^{19}F NMR spectrum of **5** in DMSO-d6

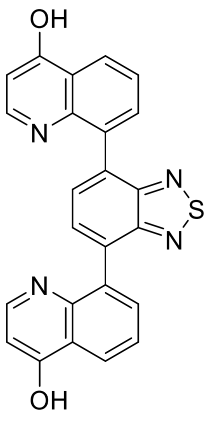

5

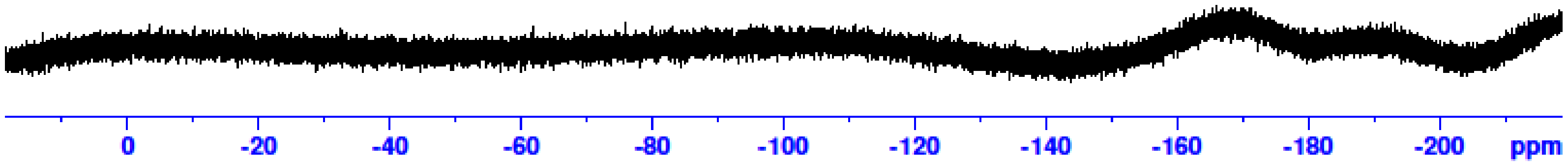

5H_2^{2+} in TFA-d

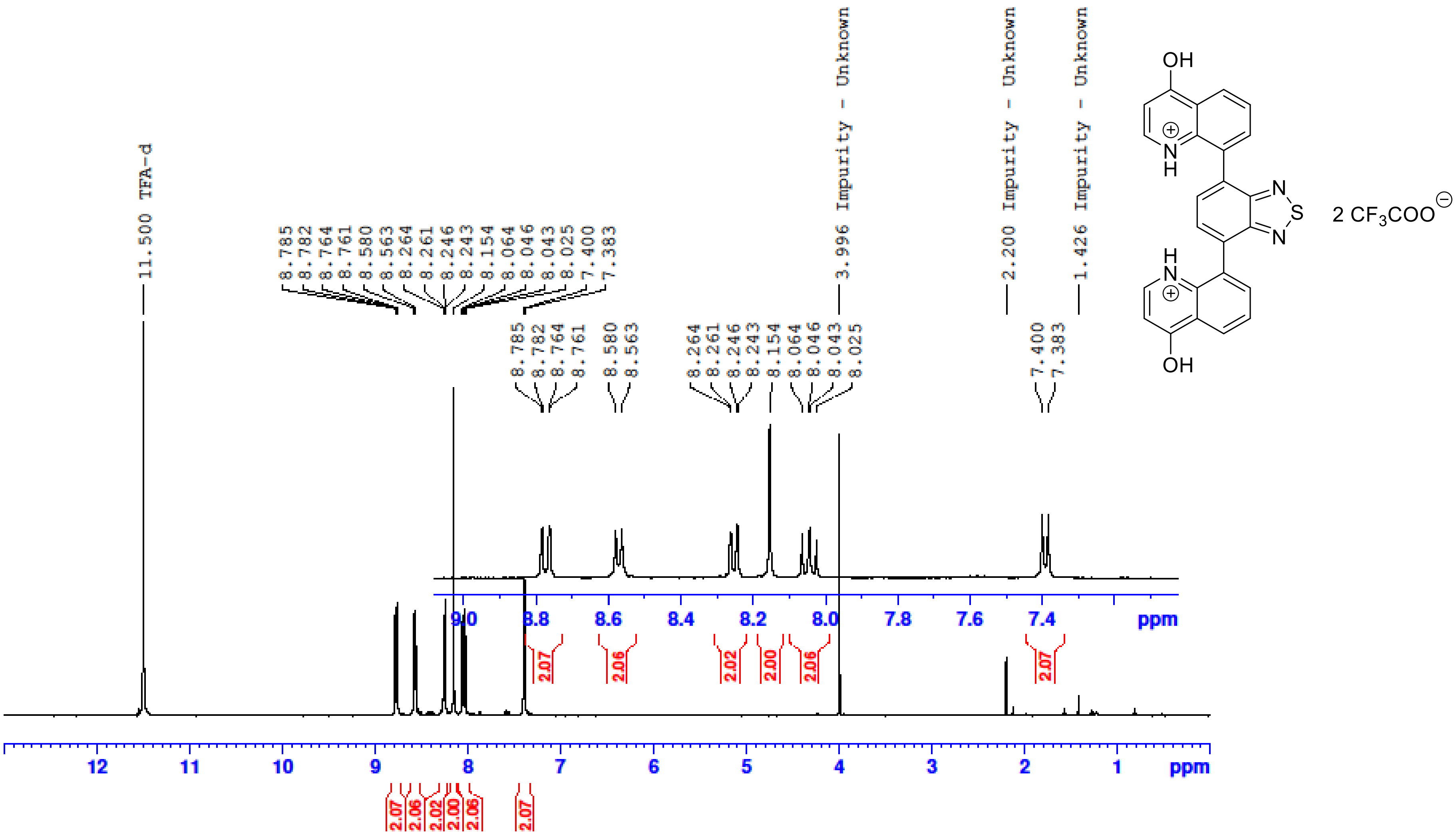

11.500 TFA-d
8.785
8.782
8.764
8.761
8.580
8.563
8.264
8.261
8.246
8.243
8.154
8.064
8.046
8.043
8.025
7.400
7.383
3.996 Impurity - Unknown
2.200 Impurity - Unknown
1.426 Impurity - Unknown
2.07
2.06
2.02
2.00
2.06
2.07
ppm
OH
N
H
N
S
N
2 CF3COO

5H_2^{2+} in TFA-d

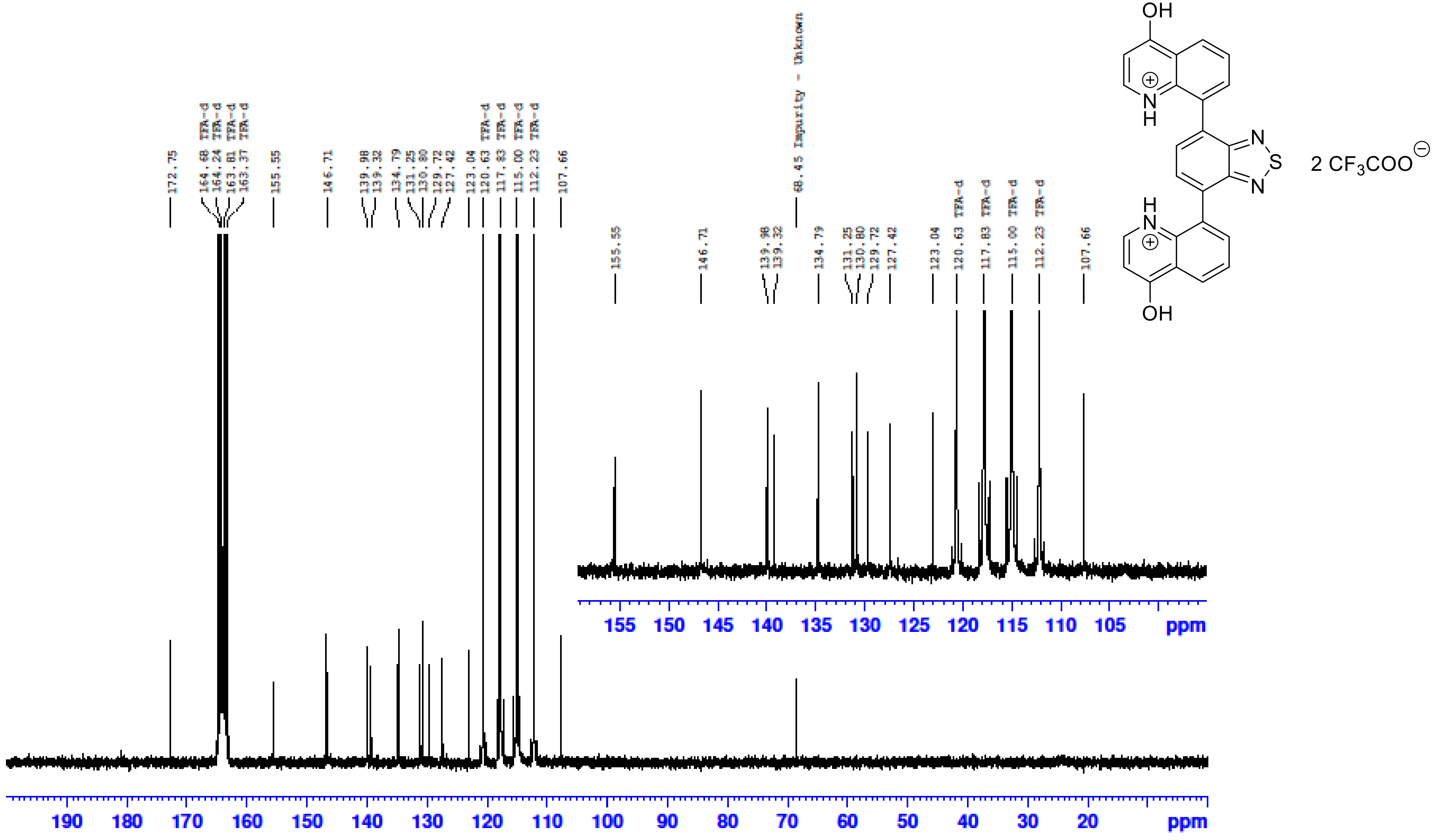

172.75
164.68 TFA-d
164.24 TFA-d
163.81 TFA-d
163.37 TFA-d
155.55
146.71
139.98
139.32
134.79
131.25
130.80
129.72
127.42
123.04
120.63 TFA-d
117.83 TFA-d
115.00 TFA-d
112.23 TFA-d
107.66
68.45 Impurity - Unknown
ppm
OH
N
H
N
S
N
H
N
OH
2 CF3COO

5H_2^{2+} in DMSO-d6

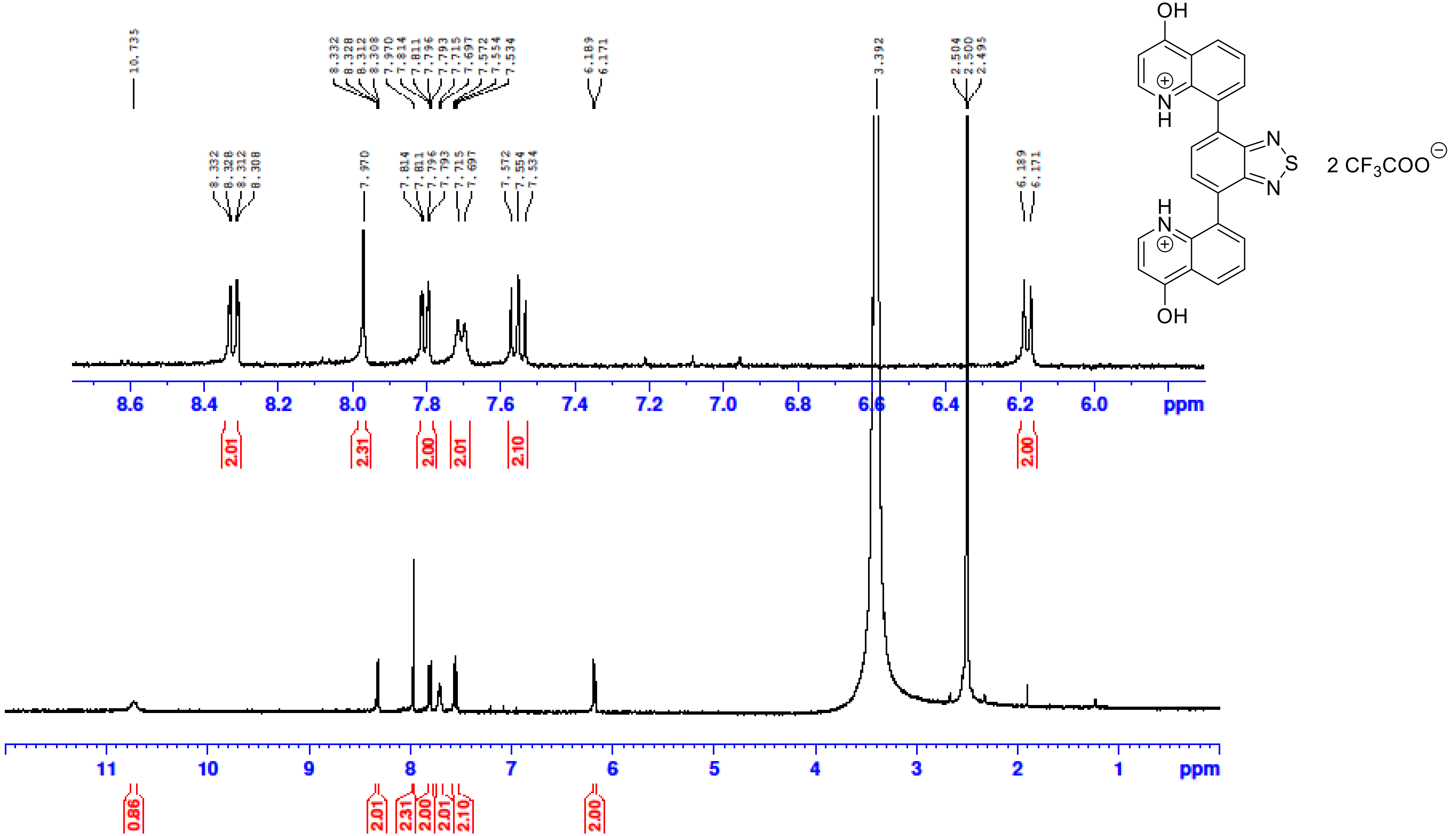

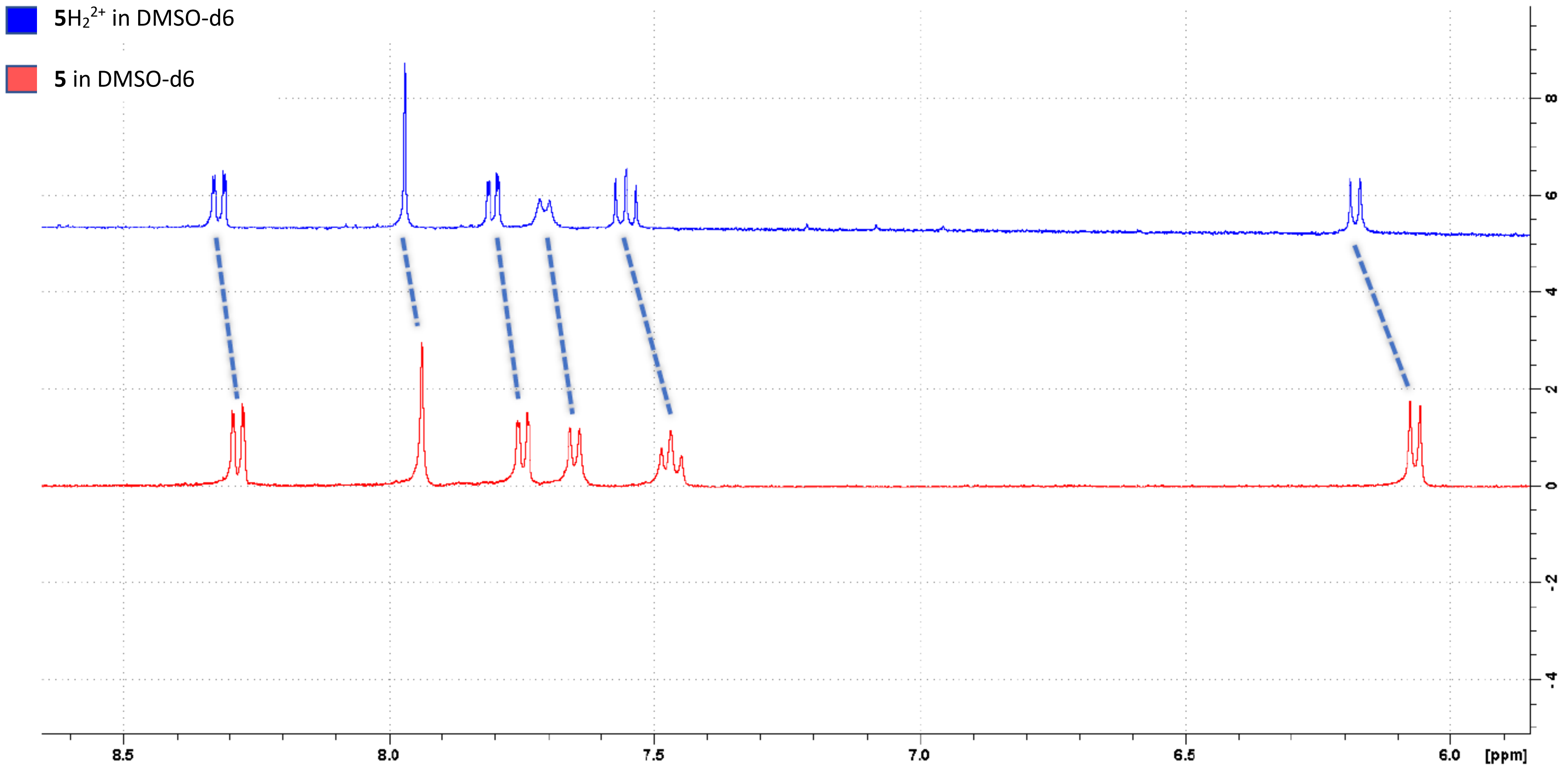

5H2 2+ in DMSO-d6
5 in DMSO-d6
8.5
8.0
7.5
7.0
6.5
6.0
[ppm]

^{19}F NMR spectrum of **5**H_2^{2+} in DMSO-d6

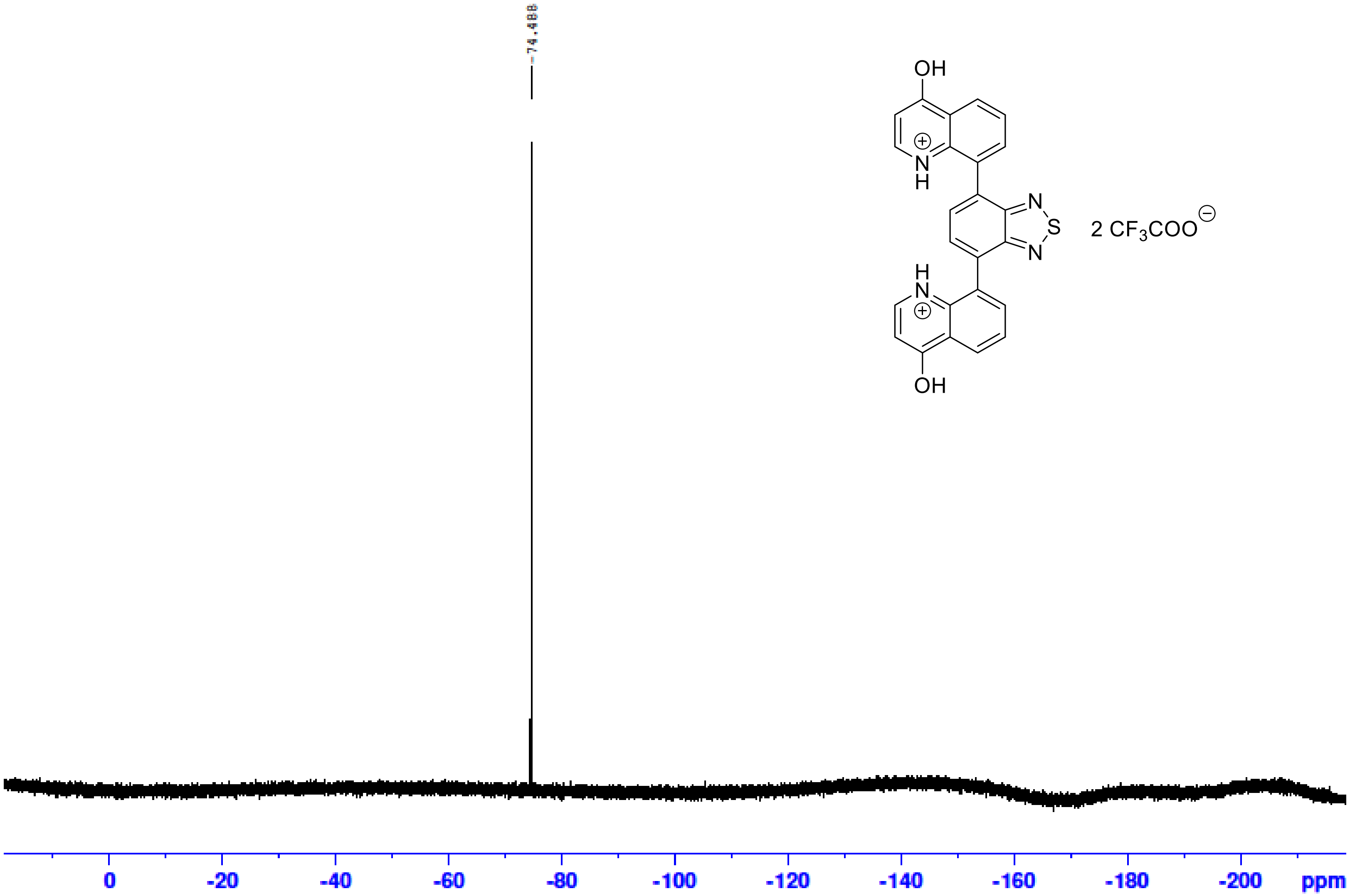

COSY spectrum of **5**H_2^{2+} in TFA-d

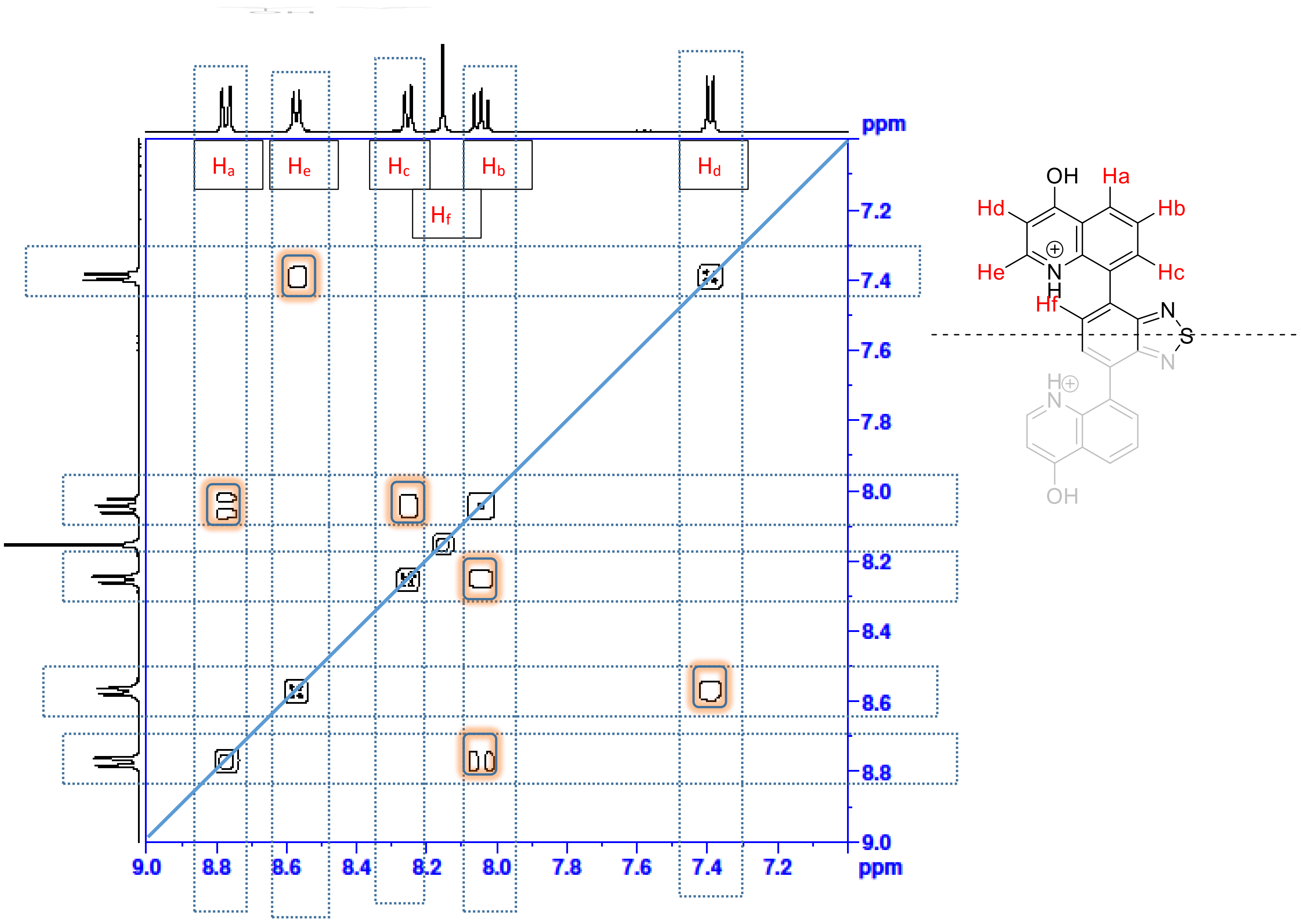

NOESY spectrum of **5**H_2^{2+} in TFA-d

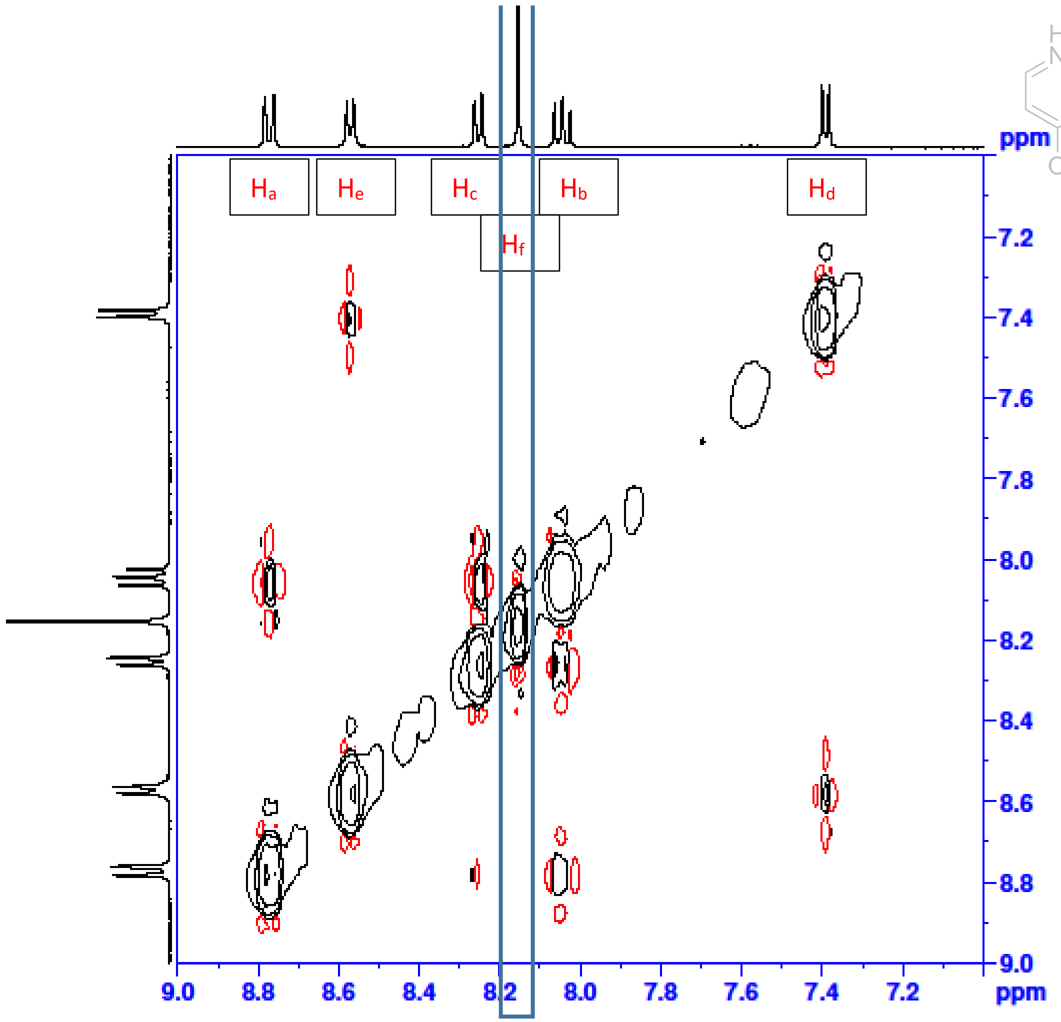

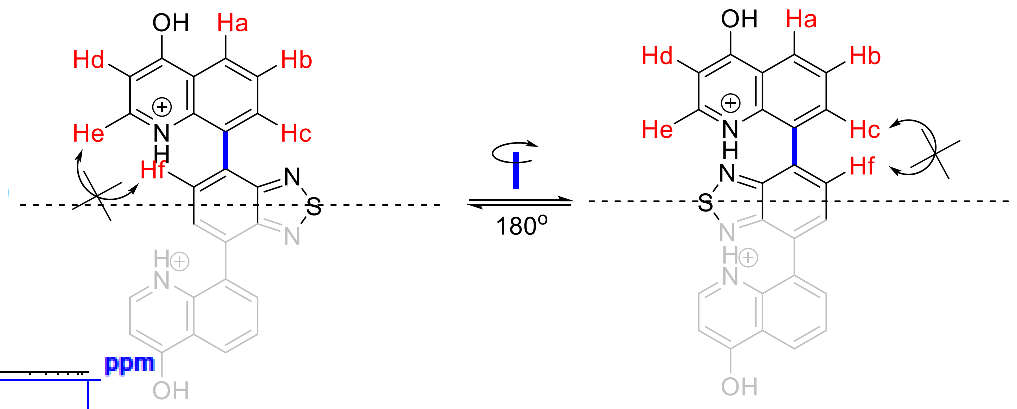

HMBC spectrum of **5**H_2^{2+} in TFA-d

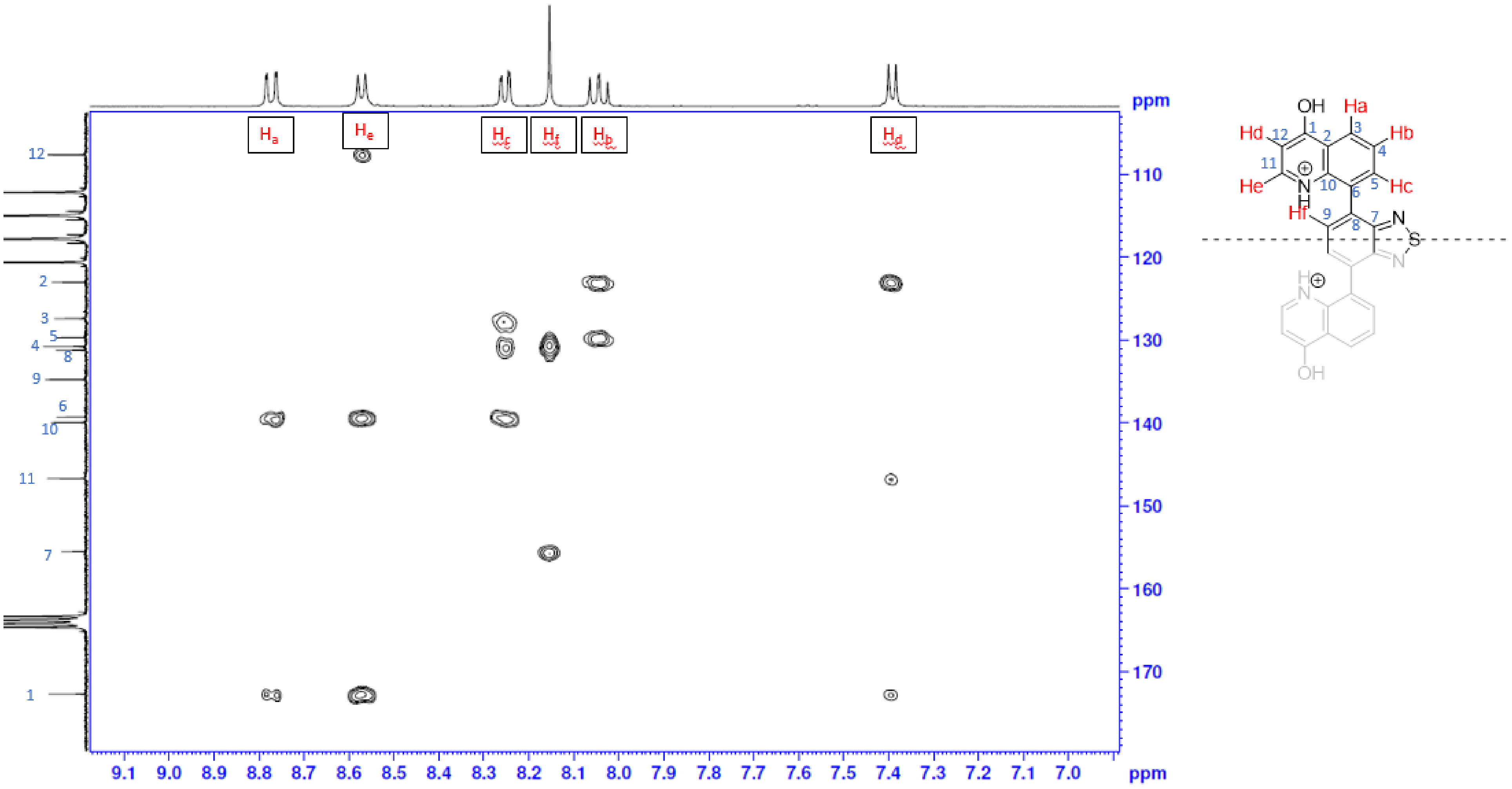

DOSY spectrum of **5**H_2^{2+} in TFA-d

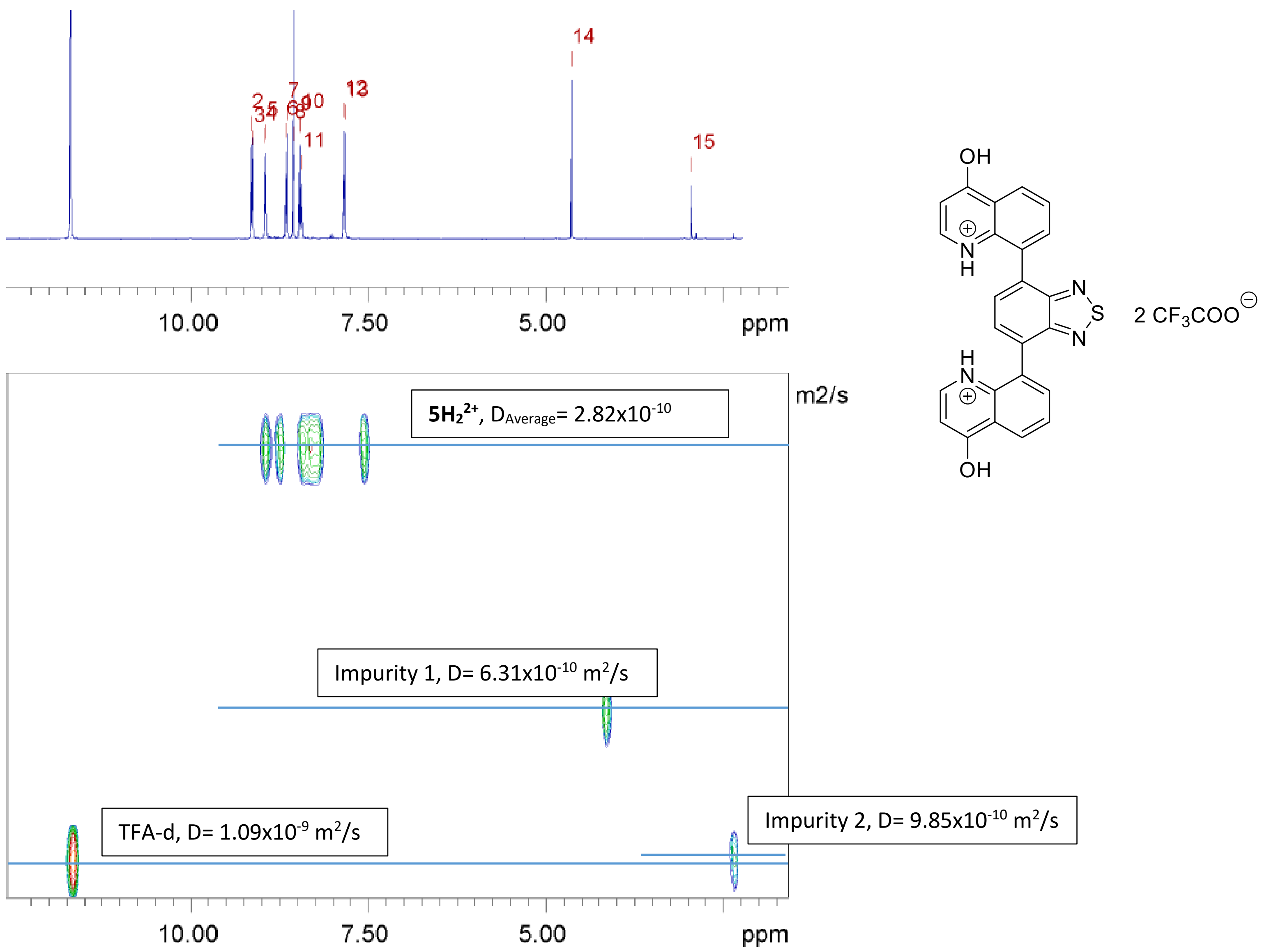

S4 MS spectra of compounds **5** and **5**H_2^{2+}

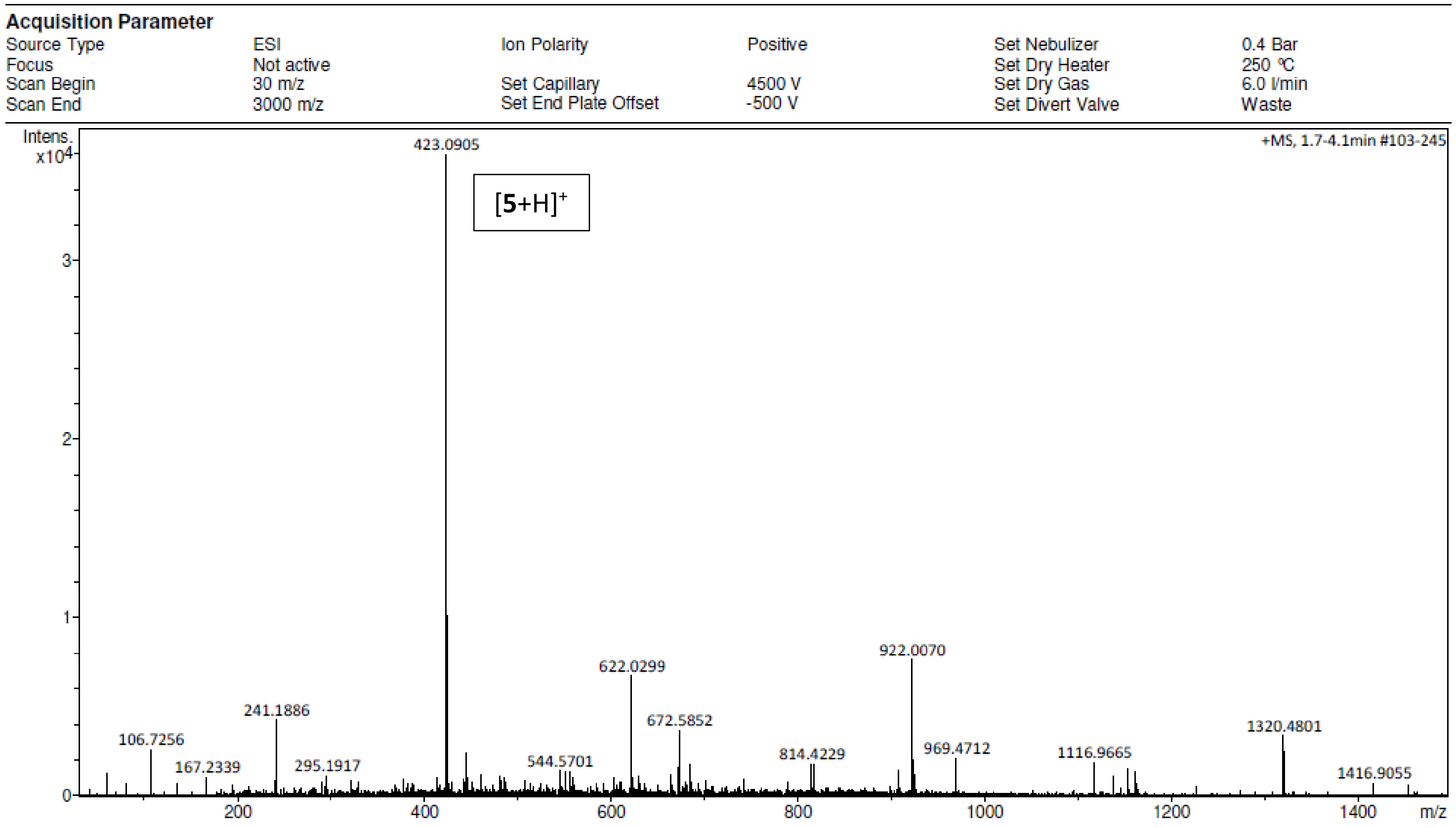

Display Report

Analysis Info

Analysis Name	D:\Data\Alans Data March 2021\HWU_Emmanouil Broumidis Cypmol 2 neg2.d	Acquisition Date	3/12/2021 10:04:31 AM
Method	010713alanstune_low.m	Operator	Bruker UK
Sample Name	Emmanouil Broumidis Cypmol 2 neg2	Instrument	micrOTOF 8213750.10408
Comment	Emmanouil Broumidis Cypmol 2 neg2		

Acquisition Parameter

Source Type	ESI	Ion Polarity	Negative	Set Nebulizer	1.0 Bar
Focus	Active			Set Dry Heater	250 °C
Scan Begin	40 m/z	Set Capillary	4500 V	Set Dry Gas	4.0 l/min
Scan End	3000 m/z	Set End Plate Offset	-500 V	Set Divert Valve	Waste

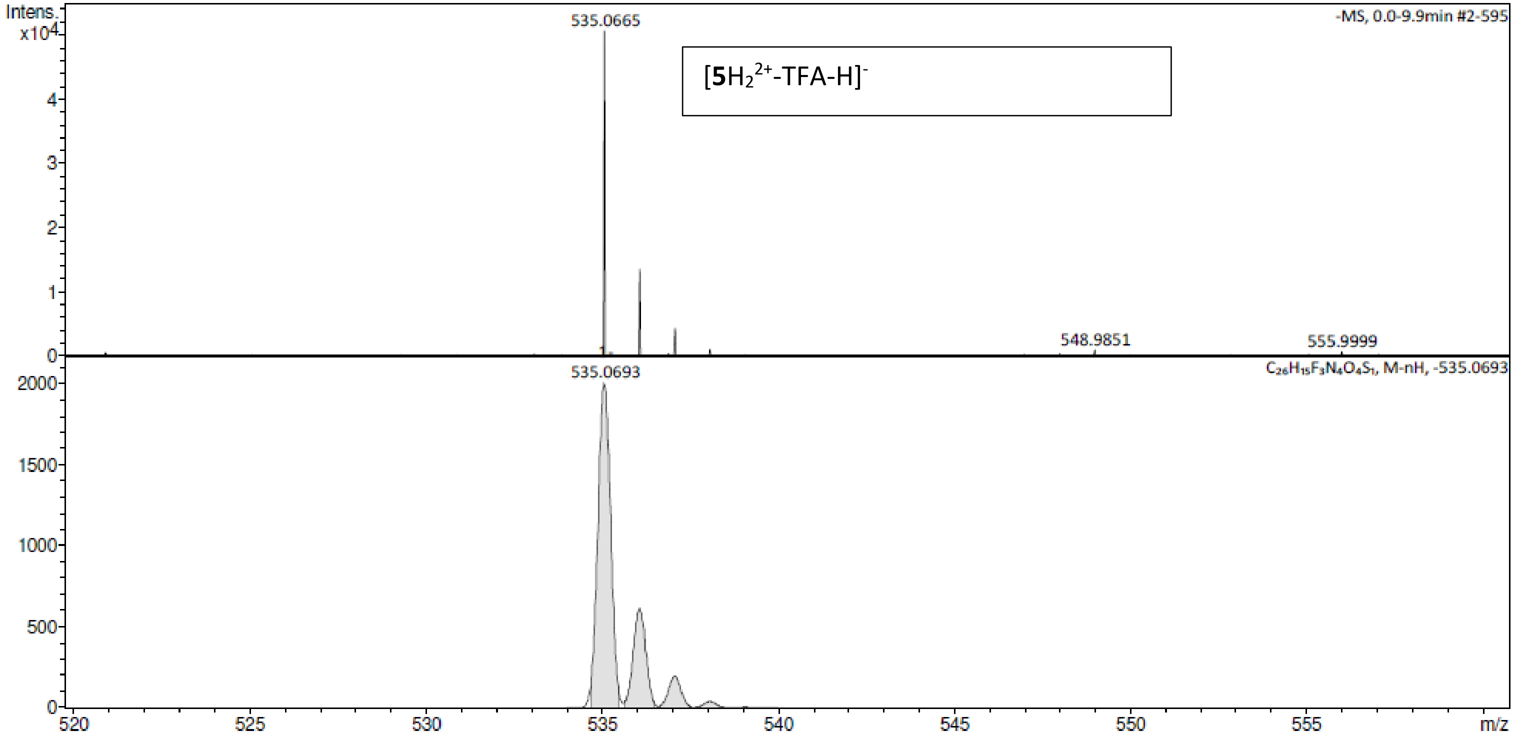

S5 References

1 E. Broumidis and P. A. Koutentis, *Tetrahedron Lett.*, 2017, **58**, 2661–2664.

2 C. G. Thomson, C. M. S. Jones, G. Rosair, D. Ellis, J. Marques-Hueso, A. L. Lee and F. Vilela, *J. Flow Chem.*, 2020, **10**, 327–345.

3 C. M. S. Jones, N. Panov, A. Skripka, J. Gibbons, F. Hesse, J.-W. G. Bos, X. Wang, F. Vetrone, G. Chen, E. Hemmer and J. Marques-Hueso, *Opt. Express*, 2020, **28**, 22803.

4 P. D. C. Reichardt, in *Solvents and Solvent Effects in Organic Chemistry*, John Wiley & Sons, Ltd, 2002, pp. 389–469.

5 Gaussian 03, Revision C.02, Frisch, M. J.; Trucks, G. W.; Schlegel, H. B.; Scuseria, G. E.; Robb, M. A.; Cheeseman, J. R.; Montgomery, Jr., J. A.; Vreven, T.; Kudin, K. N.; Burant, J. C.; Millam, J. M.; Iyengar, S. S.; Tomasi, J.; Barone, V.; Mennucci, B.; Cossi, M.; Scalmani, G.; Rega, N.; Petersson, G. A.; Nakatsuji, H.; Hada, M.; Ehara, M.; Toyota, K.; Fukuda, R.; Hasegawa, J.; Ishida, M.; Nakajima, T.; Honda, Y.; Kitao, O.; Nakai, H.; Klene, M.; Li, X.; Knox, J. E.; Hratchian, H. P.; Cross, J. B.; Bakken, V.; Adamo, C.; Jaramillo, J.; Gomperts, R.; Stratmann, R. E.; Yazyev, O.; Austin, A. J.; Cammi, R.; Pomelli, C.; Ochterski, J. W.; Ayala, P. Y.; Morokuma, K.; Voth, G. A.; Salvador, P.; Dannenberg, J. J.; Zakrzewski, V. G.; Dapprich, S.; Daniels, A. D.; Strain, M. C.; Farkas, O.; Malick, D. K.; Rabuck, A. D.; Raghavachari, K.; Foresman, J. B.; Ortiz, J. V.; Cui, Q.; Baboul, A. G.; Clifford, S.; Cioslowski, J.; Stefanov, B. B.; Liu, G.; Liashenko, A.; Piskorz, P.; Komaromi, I.; Martin, R. L.; Fox, D. J.; Keith, T.; Al-Laham, M. A.; Peng, C. Y.; Nanayakkara, A.; Challacombe, M.; Gill, P. M. W.; Johnson, B.; Chen, W.; Wong, M. W.; Gonzalez, C.; and Pople, J. A., Gaussian, Inc., Wallingford CT, 2004.
